# Barriers and facilitators influencing referral and access to palliative care for children and young people with life-limiting and life-threatening conditions: a scoping review of the evidence

**DOI:** 10.1177/02692163241271010

**Published:** 2024-09-09

**Authors:** Pru Holder, Lucy Coombes, Jane Chudleigh, Richard Harding, Lorna K Fraser

**Affiliations:** 1 Cicely Saunders Institute of Palliative Care, Policy and Rehabilitation, King’s College London, London, UK; 2 Royal Marsden NHS Foundation Trust, Sutton, Surrey, UK

**Keywords:** Child, palliative-care, terminal-care, referral-and-consultation, access-to-care

## Abstract

**Background:**

Palliative care is an essential component of children’s health services but is accessed by fewer children than could potentially benefit.

**Aim:**

Appraise the evidence to identify factors influencing referral and access to children’s palliative care, and interventions to reduce barriers and improve referrals.

**Design:**

Scoping review following the six stages of the Arksey and O’Malley framework. Data were charted using an adapted version of the socioecological framework.

**Data sources:**

CINAHL, MEDLINE, PsycINFO, EMBASE, Cochrane Library were searched for primary studies of any design and literature/systematic reviews. Studies reporting barriers/facilitators and interventions in relation to referral of children with a life-limiting condition to palliative care, in any setting, were included.

**Results:**

One hundred ninety five articles (primary qualitative and quantitative studies, reviews) were retained (153 reporting barriers/facilitators; 40 interventions; 2 both). Multiple factors were identified as barriers/facilitators: Individual level: underlying diagnosis, prognostic uncertainty, parental attitudes, staff understanding/beliefs; Interpersonal level: family support, patient-provider relationships, interdisciplinary communication; Organisational level: referral protocols, workforce, leadership; Community level: cultural norms, community resources, geography; Society level: policies and legislation, national education, economic environment, medication availability. Most of these factors were bi-directional in terms of influence. Interventions (*n* = 42) were mainly at the organisational level for example, educational programmes, screening tools/guidelines, workplace champions and new/enhanced services; one-third of these were evaluated.

**Conclusion:**

Barriers/facilitators to paediatric palliative care referral are well described. Interventions are less well described and often unevaluated. Multi-modal approaches incorporating stakeholders from all levels of the socioecological framework are required to improve paediatric palliative care referral and access.

**What is already known about this topic?**

- Only a minority of children and young people who could benefit from palliative care are referred.
- When referral to paediatric palliative care does occur, this is often late in the child or young person’s disease trajectory.
- The impact of children and young people not receiving palliative care includes unmanaged symptoms, and unaddressed emotional and psychosocial impacts of life-limiting conditions on children and their families.

**What this paper adds?**

- A synthesis of the evidence from a large number of recent papers describing barriers and facilitators to paediatric palliative care, predominantly from the US and from healthcare professional viewpoints.
- Strategies to enhance referrals to paediatric palliative care need to consider individual, interpersonal, organisational, community and societal factors.
- A need for studies developing interventions aimed at enhancing referrals to paediatric palliative care to robustly evaluate effectiveness; only one-third of studies reporting interventions evaluated their effectiveness.

**Implications for practice, theory or policy**

- An approach that considers all levels of the socioecological framework is required to improve referral and access to paediatric palliative care.
- Strategies to improve paediatric palliative care referrals need to be adapted to the context they are intended to be used in.
- Education is required to challenge the widely held perception that paediatric palliative care equates to end of life care in order for the benefits of early integration to be realised.

## Introduction

There are 21 million children and young people (hereafter ‘children’) with life-limiting or life-threatening conditions (hereafter ‘life-limiting’) worldwide that would benefit from input from palliative care services.^1,2^

Paediatric palliative care is a total and active approach to care, which aims to address symptom management, and enhance adherence to disease-orientated therapies, advance care planning, holistic family support, end of life care and bereavementt.^3–7^ Palliative care should be delivered from the point of diagnosis of a life-limiting condition in order to ensure patients and families can fully benefit from improved outcomes.^2^ However, only a small number of children who could benefit from paediatric palliative care input are referred,^8^ with many referred late.^9,10^ It is estimated that 90% of children worldwide who could benefit from specialist palliative care never receive it.^1^ The impacts of children not receiving palliative care may include poorly managed symptoms and negative emotional and psychosocial effects (e.g. isolation from peers/school and anxiety and distress) on children and their families. Without assessing and addressing these symptoms and concerns, children with life-limiting conditions may not be able to maximise their opportunities in life.^11^ Strategies are needed to improve referral and access to palliative care within health systems.^12^

Whilst there is a wealth of studies on barriers and facilitators to referral and access to paediatric palliative care, there is currently no clear overview of the existing literature. Existing reviews have focussed on specific populations (e.g. children and families^13^; health professionals^14^), conditions (e.g. oncology^15,16^), settings (e.g. community^17^; telehealth^18^), contexts (e.g. countries of high income^19–22^; countries of lower-middle income^23–26^), or barriers/facilitators (e.g. professionals delaying discussions^27^). A comprehensive synthesis of the existing evidence on all life-limiting conditions, stakeholders and contexts is necessary to inform feasible, acceptable and effective mechanisms to address the need for improved access to paediatric palliative care. The aim of this scoping review was to assess the extent and type of evidence in relation to barriers and facilitators influencing referral and access to palliative care for children with life-limiting conditions in their entirety, rather than focussing on one condition, population or context, and for interventions to reduce barriers and improve referrals.

## Methods

### Study design

A scoping review design was selected to determine the coverage of the body of literature and to identify and map the available evidence.^28^ The review was conducted following the six stages of the Arksey and O’Malley^29^ framework: (1) identifying the research question; (2) identifying relevant studies; (3) study selection; (4) charting the data; (5) collating, summarising, and reporting the results and (6) consultation with stakeholders. Recommendations by Levac et al.^30^ and the Joanna Briggs Institute^31^ were incorporated: linking the rationale of the review and research question (stage one); balancing feasibility with breadth and comprehensiveness (stage two); using an iterative team approach to selecting studies (stage three) and extracting data (stage four); considering the implications of study findings to policy, practice, or research (stage five); and incorporating consultation and knowledge transfer with stakeholders (stage six). Reporting follows the Preferred Reporting Items for Systematic Reviews and Meta-Analyses extension for Scoping Reviews (PRISMA-ScR).^32^ The protocol was registered on the Open Science Framework (https://osf.io/px38f).

Two consultation workshops with key stakeholders were held during the review process (parents of children with a life-limiting condition [*n* = 2], charity professionals across the palliative care sector [*n* = 2], paediatric palliative care consultant [*n* = 1], and children’s palliative care nurse [*n* = 1]). Potential stakeholders were identified through existing networks and contacts of the research team. During workshop one, preliminary findings from the review search were presented and feedback was sought on the review process and the collation and synthesis of the data. During workshop two, the final results of the review were presented, and feedback was sought on the interpretation of the findings.

### Search strategy

CINAHL, MEDLINE, PsycINFO, EMBASE and Cochrane Library were searched from inception to 16th March 2023. The search strategy included three main concepts: ‘children and young people’, ‘referral and access’ and ‘palliative care’. The full search strategies are reported in Supplemental File 1. ‘Children and young people’ were defined as any individual under 18 years of age in accordance with international^33^ and UK^34^ definitions of a child. As the terms ‘referral’ and ‘access’ are nuanced and dependent on the specific study and context, the concept of ‘referral and access’ was defined as encompassing the process of professionals offering palliative care, the initial introduction of palliative care services, the utilisation of palliative care (e.g. a patient accessing the palliative care services), and the actual availability of palliative care services. As provision of paediatric palliative care varies within and between countries, we defined ‘palliative care’ as care provided by palliative care services or multi-disciplinary teams with paediatric palliative care training, or services who self-identified as providing specialist paediatric palliative care in any setting. Inclusion and exclusion criteria are shown in Table 1.

**Table 1.** Inclusion and exclusion criteria.

Inclusion criteria
• Focus on influential factors (barriers and facilitators) on referral and access to palliative and end-of-life care for children and young people and/or interventions to reduce barriers and improve referrals.
• Participants are any stakeholders who provide data in relation to the referral of children and young people (aged from birth to ⩽17 years and 364 days) with a life-limiting or life-threatening condition to palliative and end-of-life care services.
• Any clinical, community or homecare setting.
• Published primary studies of any research design.
• Published literature or systematic reviews (if different data are presented to sources otherwise presented in the review).
• Published studies from any date.
• Written in English language.
Exclusion criteria
• Focus on children and young people aged >18 years or studies of a mixed population where the majority of participants do not consist of the target group or data is not reported separately.
• Articles such as case studies, case series, books, editorials, commentary or opinion pieces or conference abstracts.
• In language other than English.

Studies of a mixed population were included if (1) the majority of the participants consisted of our target group or (2) data were reported separately, in which case we reported on the disaggregation for this population (Table 1).

Retrieved articles were imported into Covidence.^35^ Titles and abstracts were screened independently by two of three reviewers (PH, JC and LC). Full text review of potentially eligible articles was independently conducted by the same three reviewers. Disagreements were resolved with a fourth reviewer (LF).

### Data extraction

Data was extracted by one reviewer (PH) using a data extraction tool adapted from the Joanna Briggs Institute template^31^ (Supplemental File 2). Data extraction of 25% of the included articles were verified by a second reviewer (LC). Disagreements were resolved with an additional reviewer (LF).

### Collating and summarising data

During data collation and synthesis, the authors concluded that the nature of the findings could best be represented and interpreted using an adapted version of the socioecological framework, where patterned behaviour is the outcome of interest.^36,37^ Extracted data were tabulated into the following levels: (1) Individual; (2) Interpersonal; (3) Organisational; (4) Community; (5) Society.^37^ Quality appraisal of papers was not conducted as per Joanne Briggs Institute guidance.^38^ National income levels were categorised using The Word Bank classifications.^39^

## Results

**Figure 1.**
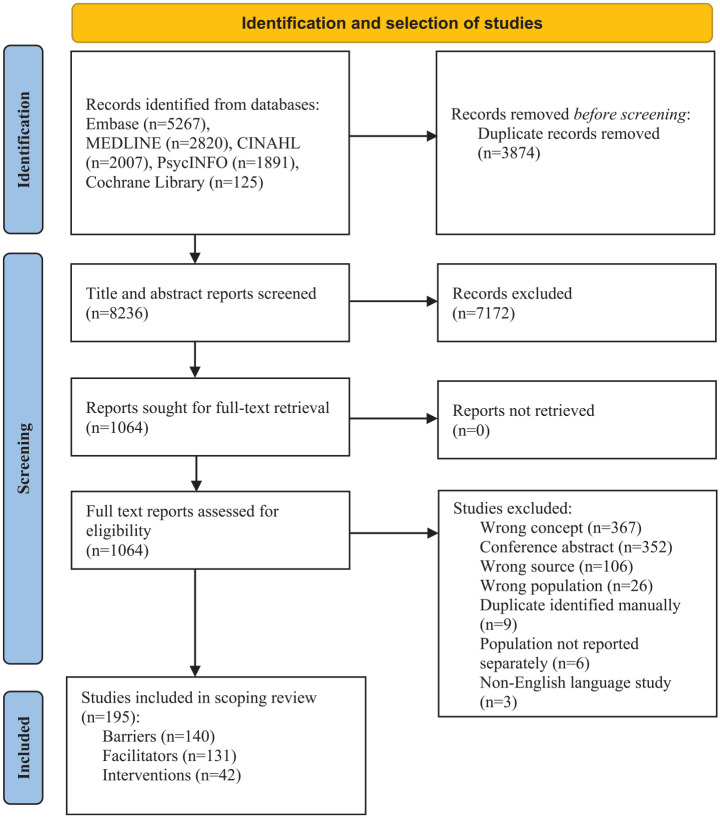
Preferred reporting items for systematic reviews and meta-analyses extension for scoping reviews flow diagram (PRISMA-ScR).^32^

Searches retrieved 12,110 articles. After deduplication, 8,236 papers were eligible for title and abstract screening and 1,064 required full text assessment. A total of 195 papers were included in the review (Figure 1). Full data extraction tables are available in Supplemental File 3.

Publication dates ranged from 2001 to 2023, with the majority published after 2010 (91%) (Table 2). Studies originated from 31 countries, with over half from North America (52%),^16,17,19,40–139^ and the remaining from Europe (14%),^22,140–166^ Asia (13%),^25,167–189^ Australia (5%),^190–198^ Africa (1%),^199,200^ South America (1%),^201,202^ or international studies (14%).^13–15,18,20,21,23,24,26,27,203–219^ Most study participants comprised health professionals (33%), or mixed stakeholders (21%). Only three studies included the views of children.^68,154,186^ The primary source of data collection was surveys (28%).

**Table 2.** Study characteristics.

Characteristic	*n* (%)
Year of publication	
2001–2009	17 (9)
2010–2019	96 (49)
2020–2023	82 (42)
Continent	
North America	103 (52)
Europe	28 (14)
International	27 (14)
Asia	24 (13)
Australia	9 (5)
Africa South	2 (1)
South America	2 (1)
Country income level	
High	156 (80)
Upper-middle	14 (8)
Lower-middle	25 (12)
Study design	
Survey	54 (28)
Interventional studies	42 (22)
Health record reviews	35 (18)
Qualitative	34 (17)
Reviews	19 (10)
Mixed methods	11 (5)
Population	
Mixed health professionals	64 (33)
Mixed stakeholders	41 (21)
Children and young people (chart review data only)	41 (21)
Clinicians	26 (13)
Nurses	15 (8)
Parents/carers/families	8 (4)
Condition/specialty	
Mixed conditions	131 (67)
Oncology	46 (23)
Cardiology	14 (7)
Neurological	3 (2)
Neurodevelopmental	1 (1)
Setting	
Hospital	87 (45)
Mixed settings	77 (39)
Pediatric/neonatal intensive care unit	22 (11)
Hospices	9 (5)

155/195 articles reported barriers and facilitators to referral and access to palliative care^13–27,40–42,44–55,57–65,68–71,73–75,79–84,86–88,90–92,96–99,103,105,107,110–115,117,118,120–127,131–134,136,138–150,153–163,165,167–169,171,173–194,196–199,201,203,205–207,211–219^ (Table 3). Contrasting barriers and facilitators were identified at each level of the of the socioecological framework; these are shown in Table 3 and described below.

**Table 3.** Key barriers and facilitators to referral and access to palliative care (*n* = 155 papers).

Level of influence from model	Examples of factors associated with increased (facilitators) and decreased (barriers) referral/access	Barriers *n* (%) of studies	Facilitators *n* (%) of studies
Individual (Patient level)	• Child’s diagnosis and impact of uncertain prognosis	40 (26)	50 (32)
	• Child’s ethnicity and cultural/linguistic factors	20 (13)	6 (4)
	• Child’s age	9 (6)	11 (7)
	• Child attitudes, treatment expectations, coping mechanisms and needs	4 (3)	2 (1)
Individual (Family level)	• Parental attitudes, treatment expectations, knowledge and experiences	55 (35)	7 (5)
	• Psychosocial aspects – parents	8 (5)	2 (1)
	• Parental financial factors	6 (4)	2 (1)
	• Parental practical factors	4 (3)	2 (1)
Individual (Professional level)	• Staff attitudes and beliefs	66 (43)	33 (21)
	• Staff understanding (perceived and actual)	58 (37)	22 (14)
	• Psychosocial aspects – staff	21 (14)	7 (5)
	• Staff demographics	11 (7)	23 (15)
Interpersonal	• Patient-provider relationship	32 (21)	23 (15)
	• Provider/interdisciplinary communication	30 (19)	15 (10)
	• Staff communication skills/information provision	28 (18)	17 (11)
	• Family support and decision making	22 (14)	13 (8)
	• Staff support	10 (6)	11 (7)
Organisational	• Workforce/workload/resources	55 (35)	24 (15)
	• Referral policies/protocols, eligibility criteria and timing of referrals	49 (32)	34 (22)
	• Characteristics of organisation	35 (23)	14 (9)
	• Interdisciplinary co-ordination within sites	29 (19)	44 (28)
	• Staff training provisions	19 (12)	8 (5)
	• Inter-service collaboration	16 (10)	12 (8)
	• Leadership, organisational culture and models of care	16 (10)	7 (5)
	• Nature of palliative care and practical support for health professionals	13 (8)	2 (1)
	• Telehealth	3 (2)	11 (7)
Community	• Effect of geography on distribution of services	31 (20)	8 (5)
	• Cultural norms/societal perceptions and awareness of palliative care	28 (18)	13 (8)
	• Community resources	9 (6)	17 (11)
	• Family workplace	4 (3)	1 (1)
Society, policy, and system	• National education and public awareness	48 (31)	29 (19)
	• Financial: national income, palliative care funding, reimbursement, cost of treatments	37 (24)	16 (10)
	• palliative care policies and legislation	23 (15)	14 (9)
	• Availability of services/advocacy	22 (14)	9 (6)
	• Medication availability	12 (8)	1 (1)
	• Service provision structure	9 (6)	4 (3)
	• Research and international collaboration	7 (5)	6 (4)
	• Health insurance	5 (3)	3 (2)

Key differences in barriers to referral and access between countries depending on national income level were identified. Key barriers prevalent in low-and middle-income countries included lack of paediatric palliative care provision or services dedicated to adults which children could access,^25,26,206^ and poor availability of symptom management medication.^26,184,203,206,213^ Key barriers in high income countries included need for improved palliative care education,^14,16,20,22,23,26,45,48,51,70,81,110,123,140,148,153,160,162,169,174,185,189,190,205,207,211,213,215^ and lack of reimbursement^20,81,86,88,148,149,160,205,211^ and insurance coverage.^16,127,145,148,160^

### Individual level factors

#### Patient level

Children with non-malignant illness^55,148,190^ or an uncertain prognosis^20,27,42,48,54,75,81,127,153,162,163,207,214^ were frequently reported as least likely to be referred to palliative care or were associated with late receipt of palliative care. Multiple, complex and/or chronic conditions,^17,44,59,60,65,113,124,155^ solid tumour or oncological diagnoses,^17,47,71,73,80,83,96,98,126,141–144,168,175,187,190,191,218^ and neuromuscular disease^17,96,98^ were frequently reported as resulting in a referral or early receipt of palliative care. Child’s age^17,19,55,59,60,73,83,92,96,98,103,111,141,143,144,191^ and ethnicity^17,58,86,96,98,141,191^ were associated with palliative care access and/or referral, although direction of influence across different age groups and ethnicities was not consistent between studies.

Children’s attitudes including refusal to access palliative care,^45^ reluctance to accept new services or healthcare providers,^13^ poor acceptance of condition or prognosis^182^ and a desire to not be defined by their condition^154^ were reported as barriers. In contrast, children’s confidence in the healthcare team^149^ and unmet needs (e.g. poor quality of life)^218^ facilitated their acceptance of palliative care.

#### Family level

Parental attitudes and treatment expectations were related to access to palliative care. Specifically, preference for curative treatment/life extending care,^16,19,49,54,74,110,127,140,146,154,156,159,171,173,174,188,218^ denial of their child’s condition/prognosis,^20,64,81,177,211^ fear of giving up,^53,149,163^ mistrust or scepticism about resources/technology,^107,131^ fear of abandonment/losing contact with healthcare services they know,^13,153,205^ poor heath knowledge/awareness/misconceptions of prognosis or palliative care^13,24,27,49,80,158,162,180,182,184,186,189,190,197,205,207^ (i.e. associating it with end of life care^68^), unrealistic expectations of treatment,^14,22,27,81,160,194,211^ and a refusal to accept a palliative care consultation^44,70^ were reported as barriers to access. Recognition/acceptance of their child’s terminal prognosis,^17,177^ openness to integrating palliative care in the course of treatment^159^ (particularly early on^16^) and a desire to reduce caregiving burden^211^ were reported as facilitating factors.

Psychosocial barriers for parents/families included anxiety,^22,64^ grief,^20,171^ distress,^16,53,149,182^ and guilt^176^ which could interfere with their decision-making capacity.^22^ Practical barriers included parental health problems and physical exhaustion,^197^ medical and technical burdens in the domestic environment,^149^ and limited family finances/financial burdens^86,162,171,190,193,197^ including issues with private insurance.^96^ Practical facilitators for families included family income,^86^ health insurance,^87^ and the release of caregiving burden when ceasing curative treatments.^180^

#### Professional level

Professional’s demographics such as role,^15,47,53,84,110,122,123,133,138,142,162,188,218^ field of specialisation,^68,79,165,215^ age,^47,86,87,122,167,189,215^ gender,^70,86^ ethnicity,^47,87,126^ and number of years in practice/since graduation^15,47,50,86,87,123,138,189^ were associated with their experience of referring or delivering palliative care. Direction of influence across these demographic categories was not consistent between studies. Lower levels of formal education^24,167^ and a lack of skills, knowledge and/or experience^16,19,22,27,42,54,71,80,107,110,126,139,162,169,185,187,190,197,203,214,217^ were reported as barriers.

Staff attitudes and beliefs such as fear of undermining parental hope and increasing parental anxiety,^40–42,45,61,63,79,86,105,110,125,196,207,214,215,218^ perceived lack of palliative care value/prioritisation of curative treatment,^20,27,44,48,50,61,75,105,111,173,178,180,181,183,184,189,190,213,218,219^ misunderstanding of what palliative care offers and/or when to offer it,^24,45,46,64,163,165^ unease/personal attitudes about dealing with death^14,48,146,160,162,178^ and negative attitudes towards opioids^26,213^ were reported as referral barriers. A favourable perception of palliative care,^40,41,46,48,53,63,111,125,146,149,162,163,165,173,188,189,216^ a sense of privilege/fulfilment in providing palliative care,^15,180,211^ and contributing to the child and family’s wellbeing^140,150^ facilitated referral.

Psychosocial barriers to professionals referring and providing palliative care included emotional response/burden,^48,125,139,140,149,156,178,180,196,219^ work stress,^14,15^ trauma,^14,146,173,183^ sense of personal failure,^140,173,211^ embarrassment,^15^ fear,^180^ anxiety,^14,64,211^ grief,^15,196,205^ poor perceived competence/confidence^15,81,91,118,122,123,140,148,149,165,184,188,207,211^ and impact on personal life.^15^ Conversely, positive coping skills such as resilience,^150^ self-care strategies,^15,123^ avoidance of negative emotions,^189^ and handling of uncertainty,^27^ as well as high perceived competence/confidence^40,48,81,118,165,184,188,203,211^ and a willingness to participate in palliative care training^149^ facilitated the referral or delivery of palliative care.

### Interpersonal level factors

#### Support for family and staff

For families, a lack of psychosocial,^71,148,158,185^ practical^82^ and bereavement support,^203,205^ as well as familial conflict/lack of inter-familial communication/social isolation^15,27,42,49,54,148,160,171,176,182,197,211^ hindered palliative care access. Emotional or practical support for parents and siblings from family, peers, social networks, community organisations or providers were reported as facilitators.^17,44,62,81,107,125,161,176,179,187,189,196^

For professionals, lack of emotional/psychosocial,^15,27,156,160,185^ bereavement^205^ and logistical support,^15^ professional counselling^145,174^ and supervision^145^ were reported as barriers. Provision of clinical supervision,^140,160^ peer support,^15^ reflective practice^205^ and consistent provision of counselling and support systems for professionals^48,81,146,189^ were facilitating factors.

#### Staff communication skills and the patient-provider relationship

The importance of effective communication (patience, compassion and a flexible and honest approach) in order to facilitate children’s access to palliative care was reported.^17,81,125,147,148,150,177,187,196,205,211^ Poor communication between families and staff (i.e. individual communication skills/style,^15,48,61,75,181,203^ anxiety/discomfort around discussing palliative care,^16,27,50,51,110,147,190,207,211^ and the withholding of information^160,177,197^ were reported as barriers. Conflict and mistrust among families and providers,^20,27,42,49,54,63,113,125,136,154,180^ as well as insufficient time available to build rapport^150^ and limiting the family’s and/or child’s involvement in palliative care discussions^13,22,159,171,176^ were reported as barriers. Greater familiarity,^140,150^ trust,^13,140,149,154,180^ rapport^161,184^ and collaboration^15,146,159,174,183,189^ between providers and families were reported as facilitating factors for referral and access. However, health professionals’ personal attachment and involvement with families could also make it difficult for them to offer or provide palliative care.^15,27,53,64,75^

#### Provider/interdisciplinary communication

Poor and fragmented communication or collaboration between providers^20,51,68,136,140,160–162,181,184,185,211^ (e.g. lack of teamwork or trust^15^), conflicting opinions or approaches,^14,48,61,75,110,113,136,177,190,207^ and exclusion of staff from decision-making^27,42,136,174,177,183^ served as barriers to the provision of palliative care. Mutual trust between teams due to repeated interactions over time,^46^ the ability to express values, opinions and beliefs,^146,189^ team problem solving/sharing of responsibility,^27,71^ and opportunities for communication/discussion^15,147,161,180,196^ facilitated provision of palliative care.

### Organisational level factors

#### Characteristics of organisation

Issues such as limited access to paediatric palliative care resources,^40,41,70,81,82,111,139,167,188,189^ bed shortages,^140,203,219^ unconducive physical environment for paediatric palliative care^14,160,174,183,184,194^ (i.e. lack of privacy for families^178,185,196^), inconsistent access to interpreters,^91,193^ and restrictive visiting hours^177^ were barriers to children’s access and referral to palliative care. Larger free-standing hospitals^17,132^ with fewer caseloads per provider, substantial financial assets, technology, paediatric facilities and medical equipment,^17^ as well as a suitable physical environment (i.e. ensuring privacy and comfort) for palliative care^48,146,177,180,193^ were associated with increased access to palliative care.

#### Interdisciplinary services, care co-ordination within sites and telehealth

A lack of or poorly integrated/underutilised palliative care services/hospice facilities within institutions was reported as a key barrier,^16,45,46,48,51,70,145,205,217^ and this was notably worse in paediatric services when compared with adult services.^79,211^ A lack of respite options,^132,190^ 24-7 access to providers,^71,161,190,197^ telephone support^71,190,219^ and home based services^145,214^ were all negatively associated with access to palliative care. Multi-disciplinary care, including integrated palliative care with paediatric-trained staff,^16,17,46,51,111,147,163,180,184^ 24-7 availability of services,^16,17,23,82,125,149,161,176,178,190^ presence of nursing coordinators^16,190^ and home care provision^17,19,23,26,111,140,205^ were reported as facilitating factors.

Telehealth was shown to both positively and negatively affect access. This included interruption to clinician-family relationships, depersonalisation of care and rushed pacing of information,^131^ as well as provider hesitancy as to whether telemedicine delivered equivalent care compared with in-person consultations.^18^ Conversely, telehealth was beneficial in creating accessible support^131,184^ (particularly in remote or rural areas^18^), enabling participant inclusion with a wider support network,^131,196^ offering timely communication,^131^ and the fostering of familiarity.^131^

#### Interagency collaboration and care and coordination

Many studies reported poor collaboration and partnerships between agencies,^16,71,107,147,190,197,207^ which could be due to limited opportunities to network,^80^ poor awareness from staff,^126,148,149^ as well as sub-optimal patient data sharing between institutions.^157^ Environments with better inter-organisational care coordination across settings including medical, social and community support agencies and services^17,21,80,81,161,178,190^ and optimised local specialist services^149^ facilitated the provision of and access to palliative care.

#### Referral policies and protocols, timing and eligibility criteria

Lack of palliative care policy/guidelines,^15,146,173,189^ consultation process guidelines,^46,111^ and standardised, objective referral criteria/screening tools for palliative care^20,24,51,74,82,121,181,184,205^ (or those that were rigid, restrictive or inconsistent^136,154,196,197^) delayed or impeded the initiation of palliative care. Standardised referral tools, guidelines, and protocols,^16,46,107,156,184,196,205^ as well as policies for automatic consultations,^110^ and the documentation of advance care plans^73,218^ facilitated referral and access.

#### Workforce, practical support for professionals, leadership, and organisational culture

Palliative care was often described as challenging, complex, and time-consuming with high logistical demands.^14,15,20,148,194,205^ This was often compounded by staff shortages^14,51,54,136,145,156,158,167,169,174,183–185,188,194,197,203,214^ (particularly palliative care experts^81,111,157,176,190,203,205,216^ or paediatric trained staff^19,80,127^), heavy workload/staff burnout,^20,71,113,139,150,154,185,189,207,215^ high staff turnover,^20,46,178^ vague roles and responsibility,^15,53,71,161,179,185^ no clear lines of authority,^15,140,178^ lack of support from management,^14,22,196,207^ staff hierarchy and structural problems,^27,107,140,160^ lack of administrative support,^136,139,178,207^ and the unavailability of an ethics committee^54,177^; all of which were barriers to palliative care. Facilitating factors to palliative care access included the presence of physician/administrator champions,^48^ the provision of adequate time to spend with families^48^ adequate staff levels,^146,197^ flexible staffing models,^160^ teamwork,^27^ specialist palliative care team/experts and programme coordinators,^71,80,84,111,136,140,148^ clear roles and responsibilities,^148,161^ strong leadership^196^ and institutional buy-in.^80,180^

#### Staff training provisions

Staff training and development needs and concerns^15,51,64,107,136,146,157,167,181,189,190,196,203^ and a lack of mentors^20^ within institutions were reported as palliative care access and/or referral barriers. Hospital enrolment for accreditation programmes,^132,178^ availability of additional training in rural areas,^19^ and presence of staff mentors^64,196^ had positive effects on staff’s experience of offering or delivering palliative care.

### Community level factors

#### Geography

Poor access to and quality of palliative care/hospice services were reported in remote, rural and low-income regions.^17,19,20,24,71,80,97,134,148,184,190,196,207^ A widespread distribution of patients^22,107,156,157,190,203^ and logistical issues/limited transport options^158,182,186,193,197^ restricted the implementation of and access to services. Palliative care tended to be more developed or easier to access in urban or metropolitan areas^23,98,212^ and areas with better transport facilities.^186^

#### Cultural norms/societal perceptions and awareness

Negative public attitudes,^107,158,189,190^ limited awareness and understanding of palliative care and its associated terminology,^16,22,25,53,71,139,148,160,182,186,190,197,199,207^ and societal stigma and prejudice^14,71,156,176,180,182,184,185,205^ (compounded by the media^194^), hindered access. Studies reported high community awareness and knowledge of palliative care,^16,21,25,71,182,205^ the re-framing of palliative care as ‘supportive care’^79^ or ‘symptom control services’,^184^ and the provision of education/information resources for communities^53,80,148,156,176^ as facilitators to access.

#### Community resources

Inconsistent availability of community-based hospice care,^81,158,197,203,217^ poor access to medication in the community,^193^ and a reduction in charitable donations^190^ (or donations predominately allocated to curative care^16^) were considered to impede palliative care access and provision. The availability of community resources and supports^16,98,176,190,196,212,217^ including mobile or ‘pop-up’ services,^21,156,161^ social media outreach,^80^ and the use of rural and remote liaison nurses to act as link between treating services and local communities^193^ were considered conducive to palliative care access.

#### Family workplace factors

Unsupportive parental workplace policies such as the lack of/limited paid sick leave,^17^ and risk of wage^182^ and job losses which had financial and insurance status implications^17,176^ were barriers to access. Supportive workplace policies such as flexible work hours and paid sick/annual leave^17^ were considered conducive to palliative care access.

### Society level factors

#### Financial factors

Low-national income,^26,206^ a lack of consistent and/or adequate funding mechanisms to establish and maintain palliative care services^16,48,71,107,157,190^ (or those which were adult-focused^190^) were barriers to palliative care availability or quality of provision. Many studies reported on the high treatment, referral and consultation costs for palliative care^71,176,184,207,214^ which were often compounded by a lack of reimbursement^20,81,86,88,148,149,160,205,211^ and insurance coverage.^16,127,145,148,160^ Factors including government and state support/funding for palliative care services,^16,80,178^ financial assistance for families,^186,190,217^ or reimbursement mechanisms^17,160^ and adequate private and public insurance coverage^16,176^ were facilitators to access.

#### Specific policies and legislation

Studies highlighted a lack of or inconsistent/restrictive national palliative care or pain management policies and regulations,^14,20,22,23,25,136,140,157,161,199,205,207,213,217^ and a limited legal framework for palliative care^71,148,162,185^ which impeded access. This was compounded by inadequate support, awareness and advocacy from policy makers.^71,156,160,217^ Studies reported the presence of state-based models, public policies/guidelines or regional standards for palliative care,^17,21,26,80,99,157,190^ legal safeguarding^149^ and the prioritisation of palliative care by policy makers^153,205^ as facilitating factors.

#### National education, core training, public awareness

A lack of formal or national palliative care education or training for professionals was reported as a key barrier^14,16,20,22,23,25,26,45,48,51,70,71,81,110,123,140,146,148,153,160,162,167,169,174,177,185,189,190,199,205,207,211,213,215,217^ and many studies reported its need and benefit.^15,16,21,25–27,53,70,71,80,107,110,148,149,153,160,161,184,189,190,196,205,215,217^

#### Service and medication availability

Essential palliative care medication availability was reported as limited,^23,26,71,184,203,205–207,213,214,217^ particularly paediatric pharmacy resources.^19^ Adequate availability of paediatric palliative care facilities in a variety of settings,^48,70,154,184^ advocacy networks^212^ and access to medication^71^ were described as facilitating factors to access.

#### Service provision structure

Access to palliative care was restricted in areas with no formal structure for provision^217^ and a lack of palliative care integration into the primary care system.^199^ Access was affected by the conditions of the healthcare system^22,23,26,149^ and was restricted in areas with a fragmented or insufficient health-care infrastructure.^16,24^ Adequate infrastructure to integrate palliative care in the health system^176^ was a facilitating factor.

#### Research and international collaboration

A lack of empirical research and evidence base for paediatric palliative care was reported in studies,^25,53,157,205^ particularly in under-resourced settings which compounded the underestimation of need.^24,26^ Research on the need for and cost effectiveness of services,^21,190,205^ integration of evidence-based care,^25^ international training collaborations with international experts,^217^ and sharing of best practices between countries^212^ were key recommendations to facilitate access to palliative care.

### Interventions

**Figure 2.**
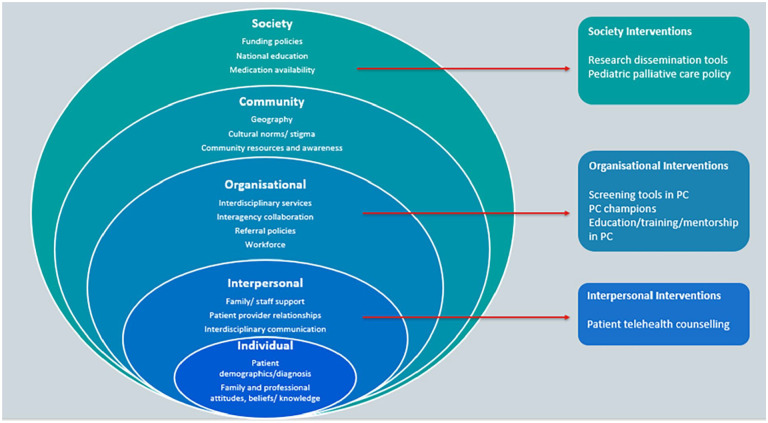
Interventions to increase access and referral to paediatric palliative care.

Interventions to improve referral to paediatric palliative care were reported by 42/195 papers^43,56,66,67,72,74,76–78,85,89,93–96,100–102,104,106,108,109,116,119,128–130,135,137,151,152,164,166,170,172,195,200,202,204,208–210^ (Figure 2 and Table 4).

**Table 4.** Interventions to increase access and referral to paediatric palliative care (*n* = 42 papers).

Level of influence from model	Examples of interventions to improve referral/access	*n* (%) Of intervention studies
Interpersonal	Telehealth counselling support	1 (2)
	Mindfulness, CBT, communication and collaboration activities for staff	1 (2)
Organisational	Education	19 (46)
	Screening/decision making tools, guidelines	11 (27)
	New/enhanced services	5 (12)
	Workplace champions	2 (5)
	Early palliative care consultations	1 (2)
Society	Research dissemination tools	1 (2)
	Paediatric palliative care policy	1 (2)

### Interpersonal level interventions

Interventions at the interpersonal level included a telehealth and counselling service delivered via a hospital-based palliative care social worker.^137^ This improved access for patients who would not otherwise have been able to receive this service. Another study used co-design sessions to consider techniques for staff for managing uncertainty and negative emotions.^74^ This was based on mindfulness and cognitive behavioural interventions, interprofessional training in palliative care communication, capacities and skills, and team collaboration activities.

### Organisational level interventions

The majority of interventions at the organisational level consisted of educational programmes.^72,77,78,93–95,108,128–130,135,152,164,172,195,200,202,209,210^ For staff this included training courses^78,93–95,108,128,129,195,210^ toolkits and workshops^164,202^ lectures,^152^ mentorship programmes,^172,200^ professional development programmes,^72,130,209^ and care curriculum development.^77,130,135^ For families, this included informational packets on palliative care.^95^ Of these 19 studies, one was unevaluated,^200^ 13 were shown to increase staff’s self-reported confidence, self-efficacy and/or knowledge in delivering palliative care,^72,77,78,94,128,135,152,164,172,195,202,209,210^ and only five evaluated the number of palliative care consultations/referrals or hospice enrolment^93,95,108,129,130^ (four of which showed an increase^93,95,108,130^).

Other studies described or evaluated new and enhanced services including the integration/expansion of paediatric palliative care programmes,^56,89^ paediatric palliative care clinics,^119^ ‘supportive care clinics’,^43^ and palliative care teams into multidisciplinary services.^166^ The integration of palliative care champions to improve the provision of paediatric palliative care were discussed in two studies,^66,106^ although neither evaluated the effectiveness of this on referrals. Other studies described the establishment and/or evaluation of referral protocols, standard recommendations, screening scales and decision-making tools for health professionals to assess palliative care needs in children,^76,85,100,116,151,170,204^ as well as standard recommendations for paediatric palliative care and consultation guidelines.^67,101,104,109^ Another study focussed on early palliative care consultations,^102^ which was shown to be acceptable to families although it was unclear if number of referrals increased.

### Society level interventions

Interventions at the society level included a paediatric palliative cancer care advocacy tool aimed at enhancing dissemination of research findings regarding palliative care integration for children with cancer, although its direct impact on access to palliative care services was not tested.^208^ Another study in the US examined the effect of a palliative care policy on hospice utilisation for children and their families residing in paediatric policy counties relative to those who did not.^96^ No effect on hospice enrolment was found, although, the policy was positively associated with length of stay in hospice care.

Of the 42 interventions reported, 17 (36%) were evaluated and either shown to positively impact receipt of paediatric palliative care or number of consultations/referrals^43,56,66,67,76,89,93,95,101,108,109,116,119,130,137^ or have no impact on number of consultations/enrolment.^96,129^ 15 (33%) were shown to improve professional’s confidence, awareness and understanding of paediatric palliative care^72,77,78,85,94,104,128,135,152,164,172,195,202,209,210^ and 10 (31%) did not measure impact on receipt of paediatric palliative care or provider experience of referring or delivering palliative care.^74,100,102,106,151,166,170,200,204,208^

## Discussion

### Main findings

This review has identified a large volume of literature describing barriers and facilitators associated with referral and access to paediatric palliative care.^13–27,40–42,44–55,57–65,68–71,73–75,79–84,86–88,90–92,96–99,103,105,107,110–115,117,118,120–127,131–134,136,138–150,153–163,165,167–169,171,173–194,196–199,201,203,205–207,211–219^ In line with the socioecological framework, which recognises that interactions between individuals and their environment are reciprocal, this review found multiple contrasting and interlinked barriers and facilitators across every level of the framework. Most of these findings come from professional views and opinions rather than direct report from parents or children themselves.

There was a much smaller body of literature describing interventions to address these barriers,^43,56,66,67,72,74,76–78,85,89,93–96,100–102,104,106,108,109,116,119,128–130,135,137,151,152,164,166,170,172,195,200,202,204,208–210^ with few evaluating impact on receipt of paediatric palliative care or number of consultations/referrals.^43,56,66,67,76,89,93,95,96,101,108,109,116,119,129,130,137^

### What this study adds

Key differences in barriers and facilitators associated with referral and access to paediatric palliative care were found between national income level. Findings prevalent in high income countries included need for improved palliative care education,^14,16,20,22,23,26,45,48,51,70,81,110,123,140,148,153,160,162,169,174,185,189,190,205,207,211,213,215^ and lack of reimbursement^20,81,86,88,148,149,160,205,211^ and insurance coverage.^16,127,145,148,160^ Most research was conducted in the US,^16,17,19,40–67,69–78,80,81,83–93,95–118,120,124–132,136–139^ a country which has a healthcare system reliant on health insurance coverage. Many healthcare plans exclude supportive services often required in paediatric palliative care such as psychosocial support and respite.^16^ Home visit provision is often capped, meaning palliative care is mainly provided in the inpatient setting.^16^ Within many other high income countries healthcare is state funded, and provision of non-medical support services and home care is more widespread. This review also highlighted issues prevalent in low- and middle-income countries. These include lack of any paediatric palliative care provision or services dedicated to adults which children could access,^25,26,206^ and poor availability of symptom management medication.^26,184,203,206,213^ Therefore, strategies to improve paediatric palliative care referrals may need to be developed within the resources available in the setting they are intended to be used in. Lower cost strategies developed with those from low- and middle-income countries may also be useful in high income countries.

Many of the barriers and facilitators to paediatric palliative care identified in this review were related to underlying diagnosis,^17,22,40,41,47,52,57,59,60,62,65,69,71,73,80,83,96,98,99,111,112,126,133,134,141–144,155,159,175,187,190–192,218^ prognostic uncertainty,^20,27,42,48,54,75,81,127,153,162,163,207,214^ and parental expectations of care outcomes such as preference for curative treatment or life extending care.^16,19,49,54,74,110,127,140,146,154,156,159,171,173,174,188,218^ Children with certain diagnoses, such as solid tumours,^17,83,175,187,218^ multiple complex or chronic conditions^17,44,59,60,65,113,124,155^ and neuromuscular disease^17,96,98^ were more likely to be referred or access palliative care, presumably due to more certainty regarding prognosis. Development of referral guidelines and policies relevant to individual settings may be beneficial to allay some of this uncertainty regarding referral. Previous research with parents of children with a cancer diagnosis identified that parents do not see curative and symptom-directed supportive care as mutually exclusive.^220^ This review found that parental openness to integrating palliative care in the course of treatment facilitated acceptance of palliative care.^159^ Our findings support this notion that children and families with life-limiting conditions should be offered a dual approach to care, whereby palliative care is received alongside disease-focussed management. Palliative care should be integrated early for all children identified as having a life-limiting condition, and parallel planning should occur.^221^ By taking this approach professionals can be less concerned with prognostic uncertainty and more children that could benefit from paediatric palliative care would receive it.

This review also found that staff beliefs and attitudes to paediatric palliative care impacted provision and access.^13–16,24,26,27,40–42,44–46,48,50,53,61,63,64,68,74,75,79,86,88,91,105,107,110,111,113,118,121–123,125,133,136,138,140,146,149,150,153,156,158,160–163,165,173,174,178,180,181,183–185,188–190,194,196,207,211,213–219^ Previous research has shown that professionals tend to equate paediatric palliative care with non-curative treatment, rather than as a dual approach to care as described above.^222^ The findings of this review support previous research that paediatric palliative care is often equated to end of life care,^53,163,222^ rather than embracing the definition that palliative care should begin at diagnosis of a life-limiting condition.^2,223^ Our findings also showed that positive health professional attitudes, including a sense of privilege/fulfilment in providing palliative care^15,180,211^ and a perception of contributing to the child and family’s wellbeing,^140,150^ facilitated the provision of palliative care. This highlights the need for improved palliative care education at an undergraduate level, as well as ongoing training for paediatric specialists to improve understanding of and attitudes towards paediatric palliative care.

Publication dates of included studies ranged from 2001 to 2023. Despite the evolution of paediatric palliative care since the early 2000s, no clear differences in barriers/facilitators by decade emerged, and barriers identified in earlier studies still persist and are relevant now. Therefore, even though paediatric palliative care services have advanced in recent years, this does not mean they are any easier to refer to or access and thus this is an area of need that still needs to be addressed.

We found a relatively small amount of literature on the development of interventions to increase referral to palliative care for children and young people across the interpersonal, organisational, and societal levels of the socioecological framework.^43,56,66,67,72,74,76–78,85,89,93–96,100–102,104,106,108,109,116,119,128–130,135,137,151,152,164,166,170,172,195,200,202,204,208–210^ Most of these interventions focussed on educational programmes and professional development^72,77,78,93–95,108,128–130,135,152,164,172,195,200,202,209,210^ and there was a clear need for more professional education regarding paediatric palliative care. Educational interventions that were evaluated were done so by asking about knowledge relatively soon after the intervention was administered, so it is not known whether this had a long-term benefit. Future studies should focus on feasibility, evaluation and implementation of interventions to ensure they are effective within the context they are intended to be used in.^224^ No interventions were identified at the individual or community levels. Future interventions could address the successes identified at these levels including positive parental, child and professional attitudes towards palliative care, improved societal awareness of palliative care and the provision of community resources.

### Strengths and limitations of the study

This review was conducted using a registered protocol and following published guidance. All identified studies were screened by two reviewers against inclusion/exclusion criteria and our stakeholder group contributed throughout the review process. Limitations of our study include inclusion of only English language articles, and potentially grey literature searching may have yielded further information. Citation searching was not conducted due to the volume of studies identified.

## Conclusions

The barriers and facilitators to referral to paediatric palliative care, especially from the views of professionals, are well described in the literature. There are fewer reports of interventions designed to increase referrals. Future research should focus on intervention development and evaluation that should understand the context in which it is to be delivered, utilise a multi-pronged approach and incorporate robust evaluation of the impact.

## Supplemental Material

sj-docx-1-pmj-10.1177_02692163241271010 – Supplemental material for Barriers and facilitators influencing referral and access to palliative care for children and young people with life-limiting and life-threatening conditions: a scoping review of the evidence Supplemental material, sj-docx-1-pmj-10.1177_02692163241271010 for Barriers and facilitators influencing referral and access to palliative care for children and young people with life-limiting and life-threatening conditions: a scoping review of the evidence by Pru Holder, Lucy Coombes, Jane Chudleigh, Richard Harding and Lorna K Fraser in Palliative Medicine

sj-docx-2-pmj-10.1177_02692163241271010 – Supplemental material for Barriers and facilitators influencing referral and access to palliative care for children and young people with life-limiting and life-threatening conditions: a scoping review of the evidence Supplemental material, sj-docx-2-pmj-10.1177_02692163241271010 for Barriers and facilitators influencing referral and access to palliative care for children and young people with life-limiting and life-threatening conditions: a scoping review of the evidence by Pru Holder, Lucy Coombes, Jane Chudleigh, Richard Harding and Lorna K Fraser in Palliative Medicine

## References

[1] Connor SR Downing J Marston J. Estimating the global need for palliative care for children: a cross-sectional analysis. J Pain Symptom Manag 2017; 53: 171–177. 10.1016/j.jpainsymman.2016.08.020 27765706

[2] Together for Short Lives. Introduction to children’s palliative care, https://www.togetherforshortlives.org.uk/changing-lives/supporting-care-professionals/introduction-childrens-palliative-care/ (2019, accessed 04 January 2023).

[3] Israëls T Kambugu J Kouya F , et al. Clinical trials to improve childhood cancer care and survival in sub-Saharan Africa. Nat Rev Clin Oncol 2013; 10: 599–604. 23897077 10.1038/nrclinonc.2013.137

[4] Chong PH De Castro Molina JA Teo K , et al. Paediatric palliative care improves patient outcomes and reduces healthcare costs: evaluation of a home-based program. BMC Palliat Care 2018; 17: 11. 29298714 10.1186/s12904-017-0267-z PMC5751774

[5] Mitchell S Morris A Bennett K , et al. Specialist paediatric palliative care services: what are the benefits? Arch Dis Child 2017; 102: 923–929. 28377450 10.1136/archdischild-2016-312026

[6] Heath JA Clarke NE McCarthy M , et al. Quality of care at the end of life in children with cancer. J Paediatr Child Health 2009; 45: 656–659. 19903251 10.1111/j.1440-1754.2009.01590.x

[7] Wolfe J Hammel JF Edwards KE , et al. Easing of suffering in children with cancer at the end of life: is care changing? J Clin Oncol 2008; 26: 1717–1723. 18375901 10.1200/JCO.2007.14.0277

[8] Craft A Killen S . Palliative care services for children and young people in England: an independent review for the secretary of state for health. London: Department of Health, 2007.

[9] Zhukovsky DS Herzog CE Kaur G , et al. The impact of palliative care consultation on symptom assessment, communication needs, and palliative interventions in pediatric patients with cancer. J Palliat Med 2009; 12: 343–349. 19327071 10.1089/jpm.2008.0152

[10] Mack JW Currie ER Martello V , et al. Barriers to optimal end-of-Life care for adolescents and young adults with cancer: bereaved caregiver perspectives. J Natl Compr Canc Netw 2021; 19: 528–533. 33571955 10.6004/jnccn.2020.7645

[11] Coombes L Braybrook D Roach A , et al. Achieving child-centred care for children and young people with life-limiting and life-threatening conditions—a qualitative interview study. Eur J Pediatr 2022; 181: 3739–3752. 35953678 10.1007/s00431-022-04566-w PMC9371630

[12] Arias-Casais N Garralda E Pons JJ , et al. Mapping pediatric palliative care development in the WHO-European region: children living in low-to-middle-income countries are less likely to access it. J Pain Symptom Manag 2020; 60: 746–753. 10.1016/j.jpainsymman.2020.04.028 32437945

[13] Mitchell S Bennett K Morris A , et al. Achieving beneficial outcomes for children with life-limiting and life-threatening conditions receiving palliative care and their families: a realist review. Palliat Med 2020; 34: 402. 10.1177/0269216319870647 PMC7074600 31431129

[14] Kain VJ. Palliative care delivery in the NICU: what barriers do neonatal nurses face? Neonatal Netw 2006; 25: 387–392. 17162999 10.1891/0730-0832.25.6.387

[15] Anastasopoulou E Dousis E. Health professionals’ views on pediatric palliative care: a mixed methods systematic review. Int J Caring Sci 2022; 15: 875–892.

[16] Haines ER Frost AC Kane HL , et al. Barriers to accessing palliative care for pediatric patients with cancer: a review of the literature. Cancer 2018; 124: 2278–2288. 29451689 10.1002/cncr.31265

[17] Boyden JY Curley MAQ Deatrick JA , et al. Factors associated with the use of U.S. community-based palliative care for children with life-limiting or life-threatening illnesses and their families: an integrative review. J Pain Symptom Manag 2018; 55: 117–131. 10.1016/j.jpainsymman.2017.04.017 28807702

[18] Miller KA Baird J Lira J , et al. The use of telemedicine for home-based palliative care for children with serious illness: a scoping review. J Pain Symptom Manag 2021; 62: 619–636.e6. 10.1016/j.jpainsymman.2020.12.004 33348029

[19] Stone W Keim-Malpass J Cozad MJ , et al. Pediatric end-of-life care in rural America: a systematic review. Am J Hosp Palliat Care 2021; 39: 1098–1104. 34963329 10.1177/10499091211064202 PMC9237176

[20] Williams-Reade J Lamson AL Knight SM , et al. Paediatric palliative care: a review of needs, obstacles and the future. J Nurs Manag 2015; 23: 4–14. 23944156 10.1111/jonm.12095

[21] Ekberg S Bowers A Bradford N , et al. Enhancing paediatric palliative care: a rapid review to inform continued development of care for children with life-limiting conditions. J Paediatr Child Health 2022; 58: 232–237. 34904760 10.1111/jpc.15851

[22] Benini F Orzalesi M de Santi A , et al. Barriers to the development of pediatric palliative care in Italy. Ann Ist Super Sanita 2016; 52: 558–564. 27999228 10.4415/ANN_16_04_16

[23] Caruso Brown AE Howard SC Baker JN , et al. Reported availability and gaps of pediatric palliative care in low- and middle-income countries: a systematic review of published data. J Palliat Med 2014; 17: 1369–1383. 25225748 10.1089/jpm.2014.0095 PMC4268583

[24] Grunauer M Mikesell C. A review of the integrated model of care: an opportunity to respond to extensive palliative care needs in pediatric intensive care units in under-resourced settings. Front Pediatr 2018; 6: 3. 29410951 10.3389/fped.2018.00003 PMC5787068

[25] Maniago JD Ngaya-An FV. Implementation science of pediatric palliative care in lower-middle-income countries in Southeast Asia: an integrative review. Pak J Med Health Sci 2021; 15: 15–2740. 10.25259/IJPC_410_20 PMC9165462 35673376

[26] Sasaki H Bouesseau M-C Marston J , et al. A scoping review of palliative care for children in low- and middle-income countries. BMC Palliat Care 2017; 16: 60. 29178866 10.1186/s12904-017-0242-8 PMC5702244

[27] Walter JK Hill DL DiDomenico C , et al. A conceptual model of barriers and facilitators to primary clinical teams requesting pediatric palliative care consultation based upon a narrative review. BMC Palliat Care 2019; 18: 116. 31864331 10.1186/s12904-019-0504-8 PMC6925857

[28] Munn Z Peters MDJ Stern C , et al. Systematic review or scoping review? Guidance for authors when choosing between a systematic or scoping review approach. BMC Med Res Methodol 2018; 18: 143. 30453902 10.1186/s12874-018-0611-x PMC6245623

[29] Arksey H O'Malley L . Scoping studies: towards a methodological framework. Int J Soc Res Methodol 2005; 8: 19–32.

[30] Levac D Colquhoun H O'Brien KK . Scoping studies: advancing the methodology. Implement Sci 2010; 5: 69. 20854677 10.1186/1748-5908-5-69 PMC2954944

[31] JBI. JBI manual for evidence synthesis, https://synthesismanual.jbi.global (2020, accessed 04 January 2023).

[32] Page MJ Moher D Bossuyt PM , et al. PRISMA 2020 explanation and elaboration: updated guidance and exemplars for reporting systematic reviews. BMJ 2021; 372: n160. 10.1136/bmj.n160 PMC8005925 33781993

[33] Assembly, UN General. Convention on the rights of the child (1989) Treaty no. 27531. United Nations Treaty Series, 1577, pp. 3–178, https://treaties.un.org/doc/Treaties/1990/09/19900902%2003-14%20AM/Ch_IV_11p.pdf (1989, accessed 04 January 2023).

[34] Children Act 1989 c.41, https://www.legislation.gov.uk/ukpga/1989/41/contents (1989, accessed 04 January 2023).

[35] Covidence systematic review software. Veritas Health Innovation. Melbourne. https://www.covidence.org/ (2023, accessed 10 January 2023).

[36] Bronfenbrenner U . The ecology of human development: experiments by nature and design. Cambridge, Massachusetts, and London, England: Harvard University Press, 1979.

[37] McLeroy KR Bibeau D Steckler A , et al. An ecological perspective on health promotion programs. Health Educ Q 1988; 15: 351–377. 3068205 10.1177/109019818801500401

[38] Joanna Briggs Institute. JBI manual for evidence synthesis, https://jbi-global-wiki.refined.site/space/MANUAL/4687770/11.3+The+scoping+review+and+summary+of+the+evidence (2023, accessed 04 January 2023).

[39] The World Bank. World bank country and lending groups, https://datahelpdesk.worldbank.org/knowledgebase/articles/906519-world-bank-country-and-lending-groups (accessed 04 January 2023).

[40] Balkin EM Kirkpatrick JN Kaufman B , et al. Pediatric cardiology provider attitudes about palliative care: a multicenter survey study. Pediatr Cardiol 2017; 38: 1324–1331. 28664445 10.1007/s00246-017-1663-0

[41] Balkin EM Sleeper LA Kirkpatrick JN , et al. Physician perspectives on palliative care for children with advanced heart disease: a comparison between pediatric cardiology and palliative care physicians. J Palliat Med 2018; 21: 773–779. 29412772 10.1089/jpm.2017.0612

[42] Bogetz JF Root MC Purser L , et al. Comparing health care provider-perceived barriers to pediatric palliative care fifteen years ago and today. J Palliat Med 2019; 22: 145–151. 30388057 10.1089/jpm.2018.0367

[43] Brock KE Allen KE Falk E , et al. Association of a pediatric palliative oncology clinic on palliative care access, timing and location of care for children with cancer. Support Care Cancer 2021; 29: 1849–1857. 32783177 10.1007/s00520-020-05671-y PMC7419028

[44] Broman A Williams C Macauley R , et al. A mixed-methods quasi-experimental study on perspectives among physicians and nurses regarding use of palliative care teams in the pediatric intensive care unit after out-of-hospital cardiac arrest. Am J Hosp Palliat Med 2021; 38: 130–137. 10.1177/1049909120937454 32638618

[45] Brown AM Nelson BH Beuscher LM. Access and barriers to utilization of palliative care in pediatric pulmonary hypertension. J Hosp Palliat Nurs 2017; 19: 474–479.

[46] Collins GS Beaman H Ho AM , et al. Perceptions of specialty palliative care and its role in pediatric stem cell transplant: a multidisciplinary qualitative study. Pediatr Blood Cancer 2022; 69: e29424. 10.1002/pbc.29424 34705322

[47] Conner NE Uddin N . Predictors of intention to refer to pediatric palliative or hospice care. Am J Hosp Palliat Care 2016; 33: 617–624. 26150677 10.1177/1049909115593062

[48] Cortezzo DE Sanders MR Brownell E , et al. Neonatologists’ perspectives of palliative and end-of-life care in neonatal intensive care units. J Perinatol 2013; 33: 731–735. 23579489 10.1038/jp.2013.38

[49] Currie ER . Parent perspectives of neonatal intensive care at the end-of-life and subsequent bereavement and coping experiences after infant death. Birmingham: University of Alabama at Birmingham, 2014.

[50] Cuviello A Raisanen JC Donohue PK , et al. Defining the boundaries of palliative care in pediatric oncology. J Pain Symptom Manag 2020; 59: 1033–1042.e1. 10.1016/j.jpainsymman.2019.11.022 PMC8979408 31838131

[51] Cuviello A Raisanen JC Donohue PK , et al. Initiating palliative care referrals in pediatric oncology. J Pain Symptom Manag 2021; 61: 81–89.e1. 10.1016/j.jpainsymman.2020.07.008 PMC9116129 32711123

[52] Cuviello A Yip C Battles H , et al. Triggers for palliative care referral in pediatric oncology. Cancers 2021; 13: 1419. DOI: 10.3390/cancers13061419. PMC8003810 33808881

[53] Dalberg T McNinch NL Friebert S. Perceptions of barriers and facilitators to early integration of pediatric palliative care: a national survey of pediatric oncology providers. Pediatr Blood Cancer 2018; 65: e26996. 10.1002/pbc.26996 29418063

[54] Davies B Sehring SA Partridge JC , et al. Barriers to palliative care for children: perceptions of pediatric health care providers. Pediatrics 2008; 121: 282–288. 18245419 10.1542/peds.2006-3153

[55] Davis ES Martinez I Hurst G , et al. Early palliative care is associated with less intense care in children dying with cancer in Alabama: a retrospective, single-site study. Cancer 2022; 128: 391–400. 34614197 10.1002/cncr.33935

[56] Davis JAM Bass A Humphrey L , et al. Early integration of palliative care in families of children with single ventricle congenital heart defects: a quality improvement project to enhance family support. Pediatr Cardiol 2020; 41: 114–122. 31676955 10.1007/s00246-019-02231-y

[57] DeCourcey DD Silverman M Oladunjoye A , et al. Patterns of care at the end of life for children and young adults with life-threatening complex chronic conditions. J Pediatr 2018; 193: 196–203.e2. 10.1016/j.jpeds.2017.09.078 PMC5794525 29174080

[58] DeGroote NP Allen KE Falk EE , et al. Relationship of race and ethnicity on access, timing, and disparities in pediatric palliative care for children with cancer. Support Care Cancer 2022; 30: 923–930. 34409499 10.1007/s00520-021-06500-6

[59] Delgado-Corcoran C Bennett EE Bodily SA , et al. Prevalence of specialised palliative care consultation for eligible children within a paediatric cardiac ICU. Cardiol Young 2021; 31: 1458–1464. 33597068 10.1017/S1047951121000433 PMC8547172

[60] Delgado-Corcoran C Wawrzynski SE Bennett EE , et al. Palliative care in children with heart disease treated in an ICU. Pediatr Crit Care Med 2020; 21: 423–429. 32142011 10.1097/PCC.0000000000002271 PMC7254981

[61] Delgado-Corcoran C Wawrzynski SE Mansfield KJ , et al. An automatic pediatric palliative care consultation for children supported on extracorporeal membrane oxygenation: a survey of perceived benefits and barriers. J Palliat Med 2022; 25: 952–957. 35319287 10.1089/jpm.2021.0452 PMC9467628

[62] Deming RS Mazzola E MacDonald J , et al. Care intensity and palliative care in chronically critically ill infants. J Pain Symptom Manag 2022; 64: 486–494. 10.1016/j.jpainsymman.2022.07.002 35840043

[63] Dickens DS. Comparing pediatric deaths with and without hospice support. Pediatr Blood Cancer 2010; 54: 746–750. 20063424 10.1002/pbc.22413

[64] Docherty SL Miles MS Brandon D. Searching for “the dying point:” providers’ experiences with palliative care in pediatric acute care. Pediatr Nurs 2007; 33: 335–341. 17907734

[65] Doorenbos AZ Starks H Bourget E , et al. Examining palliative care team involvement in automatic consultations for children on extracorporeal life support in the pediatric intensive care unit. J Palliat Med 2013; 16: 492–495. 23540309 10.1089/jpm.2012.0536 PMC3705963

[66] Drach LL Cook M Shields S , et al. Changing the culture of pediatric palliative care at the bedside. J Hosp Palliat Nurs 2021; 23: 20–27. 33136803 10.1097/NJH.0000000000000707

[67] Farooki S Olaiya O Tarbell L , et al. A quality improvement project to increase palliative care team involvement in pediatric oncology patients. Pediatr Blood Cancer 2021; 68: e28804. 10.1002/pbc.28804 33211394

[68] Fortin S Le Gall J Richer J , et al. Decision-making in the era of new medical technologies in pediatric hematology-oncology: the death of palliative care? J Pediatr Hematol Oncol 2021; 43: 271–276. 33480652 10.1097/MPH.0000000000002058

[69] Fortney CA Baughcum AE Garcia D , et al. Characteristics of critically ill infants at the end of life in the neonatal intensive care unit. J Palliat Med 2023; 26: 674–683. 36480799 10.1089/jpm.2022.0408 PMC11079611

[70] Fowler K Poehling K Billheimer D , et al. Hospice referral practices for children with cancer: a survey of pediatric oncologists. J Clin Oncol 2006; 24: 1099–1104. 16505429 10.1200/JCO.2005.02.6591

[71] Grüneberg ES Ramos-Guerrero J Pastrana T. Challenges in the provision of pediatric palliative care in Mexico: A cross-sectional Web-Based survey. J Palliat Care 2024; 39: 67. 10.1177/08258597211062767 PMC10687805 34898328

[72] Hamre TJ O'Shea ER Hinderer KA , et al. Impact of an evidence-based pediatric palliative care program on nurses’ self-efficacy. J Contin Educ Nurs 2022; 53: 264–272. 35647631 10.3928/00220124-20220505-08

[73] Harmoney K Mobley EM Gilbertson-White S , et al. Differences in advance care planning and circumstances of death for pediatric patients who do and do not receive palliative care consults: a single-center retrospective review of all pediatric deaths from 2012 to 2016. J Palliat Med 2019; 22: 1506–1514. 31233350 10.1089/jpm.2019.0111 PMC6998041

[74] Hill DL Walter JK Casas JA , et al. The codesign of an interdisciplinary team-based intervention regarding initiating palliative care in pediatric oncology. Support Care Cancer 2018; 26: 3249–3256. 29627863 10.1007/s00520-018-4190-5 PMC6379076

[75] Hill DL Walter JK Szymczak JE , et al. Seven types of uncertainty when clinicians care for pediatric patients with Advanced Cancer. J Pain Symptom Manag 2020; 59: 86–94. 10.1016/j.jpainsymman.2019.08.010 PMC6942218 31425822

[76] Humphrey L Schlegel A Seabrook R , et al. Trigger criteria to increase appropriate palliative care consultation in the neonatal intensive care unit. Pediatr Qual Saf 2019; 4: e129. 10.1097/pq9.0000000000000129 PMC6426490 30937411

[77] Jacobs HH Ferrell B Virani R , et al. Appraisal of the pediatric end-of-life nursing education consortium training program. J Pediatr Nurs 2009; 24: 216–221. 19467435 10.1016/j.pedn.2008.03.001

[78] James C Xu J Coddington J , et al. Testing a pediatric palliative care education workplace intervention. J Hosp Palliat Nurs 2022; 24: E166–E171. 10.1097/NJH.0000000000000873 35470315

[79] Jewitt N Mah K Bonares M , et al. Pediatric and adult cardiologists’ and respirologists’ referral practices to palliative care. J Pain Symptom Manag 2022; 64: 461–470. 10.1016/j.jpainsymman.2022.07.011 35905938

[80] Johnson K Allen KE West W , et al. Strengths, gaps, and opportunities: results of a statewide community needs assessment of pediatric palliative care and hospice resources. J Pain Symptom Manag 2020; 60: 512–521.e7. 10.1016/j.jpainsymman.2020.04.009 32325166

[81] Jones PM Carter BS. Pediatric palliative care: feedback from the pediatric intensivist community. Am J Hosp Palliat Care 2010; 27: 450–455. 20197556 10.1177/1049909109360410

[82] Kassam A Skiadaresis J Habib S , et al. Moving toward quality palliative cancer care: parent and clinician perspectives on gaps between what matters and what is accessible. J Clin Oncol 2013; 31: 910–915. 23182989 10.1200/JCO.2012.44.8936

[83] Kaye EC Jerkins J Gushue CA , et al. Predictors of late palliative care referral in children with cancer. J Pain Symptom Manag 2018; 55: 1550–1556. 10.1016/j.jpainsymman.2018.01.021 PMC6223026 29427739

[84] Keele L Keenan HT Bratton SL. The effect of palliative care team design on referrals to pediatric palliative care. J Palliat Med 2016; 19: 286–291. 26670933 10.1089/jpm.2015.0261

[85] Kline C Reineke A Auger J , et al. Effects of a unique pediatric hematology–oncology palliative care program on medical decision-making and communication between healthcare providers and families: results of a supportive care survey. Prog Palliat Care 2012; 20: 13–18.

[86] Knapp C Thompson L. Factors associated with perceived barriers to pediatric palliative care: a survey of pediatricians in Florida and California. Palliat Med 2012; 26: 268–274. 21680751 10.1177/0269216311409085

[87] Knapp C Thompson L Madden V , et al. Paediatricians’ perceptions on referrals to paediatric palliative care. Palliat Med 2009; 23: 418–424. 19251829 10.1177/0269216309102618

[88] Knapp CA Madden V Wang H , et al. Pediatric nurses’ attitudes toward hospice and pediatric palliative care. Pediatr Nurs 2011; 37: 121–126. 21739743

[89] Knapp CA Madden VL Curtis CM , et al. Partners in care: together for kids: Florida’s model of pediatric palliative care. J Palliat Med 2008; 11: 1212–1220. 19021484 10.1089/jpm.2008.0080

[90] Knoll C Kaufman B Chen S , et al. Palliative care engagement for pediatric ventricular assist device patients: a single-center experience. ASAIO J 2020; 66: 929–932. 32740354 10.1097/MAT.0000000000001092

[91] Kolmar A Kamal AH Steinhauser KE. Clinician end-of-life experiences with pediatric Muslim patients at a US quaternary care center. J Pain Symptom Manag 2022; 63: 673–679. 10.1016/j.jpainsymman.2022.01.005 35032621

[92] Labudde EJ DeGroote NP Ebelhar J , et al. Evaluating palliative opportunities across the age spectrum in children and adolescent patients with cancer. J Adolesc Young Adult Oncol 2022; 11: 402–409. 34582272 10.1089/jayao.2021.0081

[93] Lafond D Perko K Fisher D , et al. Impact of pediatric primary palliative care education and mentoring in practice. J Hosp Palliat Nurs 2022; 24: 22–29. 34550914 10.1097/NJH.0000000000000802

[94] Lalloo C Osei-Twum J-A Rapoport A , et al. Pediatric project ECHO®: a virtual community of practice to improve palliative care knowledge and self-efficacy among interprofessional health care providers. J Palliat Med 2021; 24: 1036–1044. 33326309 10.1089/jpm.2020.0496 PMC8215401

[95] Liberman DB Song E Radbill LM , et al. Early introduction of palliative care and advanced care planning for children with complex chronic medical conditions: a pilot study. Child Care Health Dev 2016; 42: 439–449. 27028099 10.1111/cch.12332

[96] Lindley LC. The effect of pediatric palliative care policy on hospice utilization among California Medicaid beneficiaries. J Pain Symptom Manag 2016; 52: 688–694. 10.1016/j.jpainsymman.2016.05.019 PMC5107325 27693902

[97] Lindley LC Edwards SL. Geographic access to hospice care for children with cancer in Tennessee, 2009 to 2011. Am J Hosp Palliat Care 2014; 32: 849–854. 25028742 10.1177/1049909114543641 PMC4294986

[98] Lindley LC Shaw S-L. Who are the children using hospice care? J Spec Pediatr Nurs 2014; 19: 308–315. 25131751 10.1111/jspn.12085 PMC4490584

[99] Lindley LC Trujillo LV. End-of-Life care for Hispanic children: a study of California Medicaid beneficiaries. Hisp Health Care Int 2016; 14: 164–169. 27650201 10.1177/1540415316670900 PMC5136309

[100] Lowenstein S Macauley R Perko K , et al. Provider perspective on the role of palliative care in hypoplastic left heart syndrome. Cardiol Young 2020; 30: 377–382. 32146916 10.1017/S1047951120000128

[101] Lutmer JE Humphrey L Kempton TM , et al. Screening criteria improve access to palliative care in the PICU. Pediatr Crit Care Med 2016; 17: e335–e342. 10.1097/PCC.0000000000000848 27367043

[102] Mahmood LA Casey D Dolan JG , et al. Feasibility of early palliative care consultation for children with high-risk malignancies. Pediatr Blood Cancer 2016; 63: 1419–1422. 27148856 10.1002/pbc.26024

[103] Marc-Aurele KL Hull AD Jones MC , et al. A fetal diagnostic center’s referral rate for perinatal palliative care. Ann Palliat Med 2018; 7: 177–185. 28595435 10.21037/apm.2017.03.12

[104] Mikal M Gandhi R Walsh SM. Utilization of a prenatal palliative care consultation pathway for congenital heart disease. J Hosp Palliat Nurs 2023; 25: 24–30. 36622312 10.1097/NJH.0000000000000917

[105] Morell E Thompson J Rajagopal S , et al. Congenital cardiothoracic surgeons and palliative care: a national survey study. J Palliat Care 2021; 36: 17–21. 31597507 10.1177/0825859719874765

[106] Moynihan KM Snaman JM Kaye EC , et al. Integration of pediatric palliative care into cardiac intensive care: a champion-based model. Pediatrics 2019; 144: e20190160. DOI: doi: 10.1542/peds.2019-0160. 10.1542/peds.2019-0160 PMC6855829 31366685

[107] Murday P Downing K Gaab E , et al. A qualitative study describing pediatric palliative care in non-metropolitan areas of Illinois. Am J Hosp Palliat Care 2022; 39: 18–26. 33764190 10.1177/10499091211005700

[108] Newton K Sebbens D. The impact of provider education on pediatric palliative care referral. J Pediatr Health Care 2020; 34: 99–108. 31590995 10.1016/j.pedhc.2019.07.007

[109] Nguyen L Cooperberg D Spear M. Introduction of triggers for palliative care consultation improve utilization and satisfaction within a level IV NICU (SA508D). J Pain Symptom Manag 2018; 55: 643. 10.1038/s41372-018-0067-1 29740184

[110] Niehaus JZ Palmer MM Slaven J , et al. Neonatal palliative care: perception differences between providers. J Perinatol 2020; 40: 1802–1808. 32661367 10.1038/s41372-020-0714-1

[111] Parisio KN Levy CD Lewis AM , et al. Attitudes and practices of pediatric oncologists regarding palliative care consultation for pediatric oncology patients. J Pediatr Hematol Oncol 2022; 44: 230–236. 35041359 10.1097/MPH.0000000000002276

[112] Pierucci RL Kirby RS Leuthner SR. End-of-life care for neonates and infants: the experience and effects of a palliative care consultation service. Pediatrics 2001; 108: 653–660. 11533332 10.1542/peds.108.3.653

[113] Richards CA Starks H O'Connor MR , et al. When and why do neonatal and pediatric critical care physicians consult palliative care? Am J Hosp Palliat Care 2017; 35: 840–846. 29179572 10.1177/1049909117739853 PMC5807228

[114] Roberts HJ Wang Y Spruit JL , et al. The impact of clinical trial enrollment on specialty palliative care utilization in pediatric patients with high-grade gliomas. Pediatr Blood Cancer 2023; 70: e30115. 10.1002/pbc.30115 36458446

[115] Seddighzadeh RP Lawrence K Hamby T , et al. Influence of palliative care on medical treatment of pediatric patients with complex chronic diseases at cook children’s medical center. J Palliat Med 2018; 21: 1617–1620. 30070934 10.1089/jpm.2018.0079

[116] Shaw R Seegal H Miller JG , et al. Pilot of a pediatric palliative care early intervention instrument. J Hosp Palliat Nurs 2018; 20: 486–491. 30188443 10.1097/NJH.0000000000000466

[117] Shaw T Berkel C Bernatavicius W , et al. “If we build it, will they come?” A cohort study of family utilization of a pediatric-specific hospice home. J Palliat Med 2022; 25: 1361–1366. 35363045 10.1089/jpm.2021.0513

[118] Sheetz MJ Bowman M-AS . Pediatric palliative care: an assessment of physicians’ confidence in skills, desire for training, and willingness to refer for end-of-life care. Am J Hosp Palliat Care 2008; 25: 100–105. 18445861 10.1177/1049909107312592

[119] Siden H Straatman L Miller T , et al. The Madison clinic: evaluation of a collaborative outpatient paediatric palliative care clinic. Paediatr Child Health 2009; 14: 379–384. 20592973 PMC2735380

[120] Spraker-Perlman HL Tam RP Bardsley T , et al. The impact of pediatric palliative care involvement in the care of critically ill patients without complex chronic conditions. J Palliat Med 2019; 22: 553–556. 30589623 10.1089/jpm.2018.0469

[121] St-Laurent-Gagnon T Carnevale FA Duval M. Pediatric palliative care: a qualitative study of physicians’ perspectives in a tertiary care university hospital. J Palliat Care 2008; 24: 26–30. 18459594

[122] Stenekes S Penner JL Harlos M , et al. Development and implementation of a survey to assess health-care provider's competency, attitudes, and knowledge about perinatal palliative care. J Palliat Care 2019; 34: 151–159. 30060727 10.1177/0825859718790627

[123] Straatman L Miller T. Paediatric palliative care: a survey of paediatricians and family practitioners. BMJ Support Palliat Care 2013; 3: 366–371. 10.1136/bmjspcare-2011-000058 24644757

[124] Stutz M Kao RL Huard L , et al. Associations between pediatric palliative care consultation and end-of-life preparation at an academic medical center: a retrospective EHR analysis. Hosp Pediatrics 2018; 8: 162–167. 10.1542/hpeds.2017-0016 PMC7098707 29436391

[125] Szymczak JE Schall T Hill DL , et al. Pediatric oncology providers’ perceptions of a palliative care service: the influence of emotional esteem and emotional labor. J Pain Symptom Manag 2018; 55: 1260–1268. 10.1016/j.jpainsymman.2018.01.019 PMC5908218 29425881

[126] Thompson LA Knapp C Madden V , et al. Pediatricians’ perceptions of and preferred timing for pediatric palliative care. Pediatrics 2009; 123: e777–e782. 10.1542/peds.2008-2721 19403469

[127] Varela AMS Deal AM Hanson LC , et al. Barriers to hospice for children as perceived by hospice organizations in North Carolina. Am J Hosp Palliat Care 2012; 29: 171–176. 21712308 10.1177/1049909111412580

[128] Vesel T Beveridge C. From fear to confidence: changing providers’ attitudes about pediatric palliative and hospice care. J Pain Symptom Manag 2018; 56: 205–212.e3. 10.1016/j.jpainsymman.2018.03.019 29621556

[129] Walter JK Hill DL Schall TE , et al. An interprofessional team-based intervention to address barriers to initiating palliative care in pediatric oncology: a multiple-method evaluation of feasibility, acceptability, and Impact. J Pain Symptom Manag 2021; 62: 1135–1144. 10.1016/j.jpainsymman.2021.06.008 PMC8648922 34153461

[130] Weaver MS Jenkins R Wichman C , et al. Sowing across a state: development and delivery of a grassroots pediatric palliative care nursing curriculum. J Palliat Care 2021; 36: 22–28. 31771423 10.1177/0825859719889700

[131] Weaver MS Neumann ML Navaneethan H , et al. Human Touch via touchscreen: rural nurses’ experiential perspectives on telehealth use in pediatric hospice care. J Pain Symptom Manag 2020; 60: 1027–1033. 10.1016/j.jpainsymman.2020.06.003 PMC7276120 32525081

[132] Weaver MS Shostrom VK Kaye EC , et al. Palliative care programs in children’s Hospitals. Pediatrics 2022; 150: e2022057872. DOI: 10.1542/peds.2022-057872. 36093621

[133] Wentlandt K Krzyzanowska MK Swami N , et al. Referral practices of pediatric oncologists to specialized palliative care. Support Care Cancer 2014; 22: 2315–2322. 24671435 10.1007/s00520-014-2203-6

[134] Widger K Sutradhar R Rapoport A , et al. Predictors of specialized pediatric palliative care involvement and impact on patterns of end-of-life care in children with cancer. J Clin Oncol 2018; 36: 801–807. 29356606 10.1200/JCO.2017.75.6312

[135] Widger K Wolfe J Friedrichsdorf S , et al. National Impact of the EPEC-pediatrics enhanced train-the-trainer model for delivering education on pediatric palliative care. J Palliat Med 2018; 21: 1249–1256. 29782212 10.1089/jpm.2017.0532

[136] Williams-Reade J Lamson AL Knight SM , et al. The clinical, operational, and financial worlds of neonatal palliative care: a focused ethnography. Palliat Support Care 2015; 13: 179–186. 24168724 10.1017/S1478951513000916

[137] Winegard B Miller EG Slamon NB. Use of telehealth in pediatric palliative care. Telemed J E Health 2017; 23: 938–940. 28486031 10.1089/tmj.2016.0251

[138] Wool C. Clinician confidence and comfort in providing perinatal palliative care. J Obstet Gynecol Neonatal Nurs 2013; 42: 48–58. 10.1111/j.1552-6909.2012.01432.x 23181399

[139] Wool C. Clinician perspectives of barriers in perinatal palliative care. MCN Am J Matern Child Nurs 2015; 40: 44–50. 25503834 10.1097/NMC.0000000000000093

[140] Bergsträsser E Cignacco E Luck P. Health care professionals’ experiences and needs when delivering end-of-life care to children: a qualitative study. Palliat Care 2017; 10: e1178224217724770. 10.1177/1178224217724770 PMC5555493 28835736

[141] Fraser LK Fleming T Miller M , et al. Palliative care discharge from paediatric intensive care units in Great Britain. Palliat Med 2010; 24: 608–615. 20233896 10.1177/0269216310364200

[142] Fraser LK Miller M McKinney PA , et al. Referral to a specialist paediatric palliative care service in oncology patients. Pediatr Blood Cancer 2011; 56: 677–680. 21298761 10.1002/pbc.22667

[143] Fraser LK van Laar M Miller M , et al. Does referral to specialist paediatric palliative care services reduce hospital admissions in oncology patients at the end of life? Br J Cancer 2013; 108: 1273–1279. 23449361 10.1038/bjc.2013.89 PMC3619259

[144] Friedel M Gilson A Bouckenaere D , et al. Access to paediatric palliative care in children and adolescents with complex chronic conditions: a retrospective hospital-based study in Brussels, Belgium. BMJ Paediatr Open 2019; 3: e000547. 10.1136/bmjpo-2019-000547 PMC6782038 31646199

[145] Friedrichsdorf SJ Menke A Brun S , et al. Status quo of palliative care in pediatric oncology—a nationwide survey in Germany. J Pain Symptom Manag 2005; 29: 156–164. 10.1016/j.jpainsymman.2004.05.004 15733807

[146] Girgin BA Gözen D Aktaş E , et al. Attitudes toward neonatal palliative care among Turkish nurses and physicians: a comparative cross-sectional study. J Hosp Palliat Nurs 2022; 24: E185–E196. 10.1097/NJH.0000000000000875 35470317

[147] Guerin S Kiernan G Courtney E , et al. Integration of palliative care in services for children with life-limiting neurodevelopmental disabilities and their families: a Delphi study. BMC Health Serv Res 2020; 20: 927. 33032605 10.1186/s12913-020-05754-w PMC7545942

[148] Jünger S Pastrana T Pestinger M , et al. Barriers and needs in paediatric palliative home care in Germany: a qualitative interview study with professional experts. BMC Palliat Care 2010; 9: 10. 20525166 10.1186/1472-684X-9-10 PMC2898764

[149] Jünger S Vedder AE Milde S , et al. Paediatric palliative home care by general paediatricians: a multimethod study on perceived barriers and incentives. BMC Palliat Care 2010; 9: 11. 20525318 10.1186/1472-684X-9-11 PMC2902453

[150] Kiernan G Hurley F Price J. With every fibre of their being’: Perspectives of healthcare professionals caring for children with non-malignant life-limiting conditions. Child Care Health Dev 2022; 48: 250–258. 34753200 10.1111/cch.12923

[151] Lazzarin P Giacomelli L Terrenato I , et al. A tool for the evaluation of clinical needs and eligibility to pediatric palliative care: the validation of the ACCAPED scale. J Palliat Med 2021; 24: 205–210. 32640899 10.1089/jpm.2020.0148

[152] Meglič J Lisec A Lepej D , et al. Challenges in establishing optimal pediatric palliative care at the university hospital in Slovenia. Eur J Pediatr 2023; 182: 1393–1401. 36680577 10.1007/s00431-023-04806-7 PMC10023644

[153] Mitchell S Slowther AM Coad J , et al. Facilitators and barriers to the delivery of palliative care to children with life-limiting and life-threatening conditions: a qualitative study of the experiences and perceptions of healthcare professionals. Arch Dis Child 2022; 107: 59–64. 33980510 10.1136/archdischild-2021-321808

[154] Mitchell S Slowther AM Coad J , et al. Experiences of healthcare, including palliative care, of children with life-limiting and life-threatening conditions and their families: a longitudinal qualitative investigation. Arch Dis Child 2021; 106: 570–576. 33199300 10.1136/archdischild-2020-320189 PMC8142456

[155] Nogueira A Correia D Loureiro M , et al. The needs of children receiving end of life care and the impact of a paediatric palliative care team: a retrospective cohort study. Eur J Pediatr 2022; 182: 525–531. 36445514 10.1007/s00431-022-04683-6

[156] Pacurari N De Clercq E Dragomir M , et al. Challenges of paediatric palliative care in Romania: a focus groups study. BMC Palliat Care 2021; 20: 178. 34794399 10.1186/s12904-021-00871-7 PMC8598931

[157] Papworth A Fraser L Taylor J. Development of a managed clinical network for children’s palliative care – a qualitative evaluation. BMC Palliat Care 2021; 20: 20. 33482795 10.1186/s12904-021-00712-7 PMC7824916

[158] Pentaris P Papadatou D Jones A , et al. Palliative care professional’s perceptions of barriers and challenges to accessing children’s hospice and palliative care services in South East London: A preliminary study. Death Stud 2018; 42: 649–657. 29393840 10.1080/07481187.2018.1430081

[159] Rost M Acheson E Kühne T , et al. Palliative care in Swiss pediatric oncology settings: a retrospective analysis of medical records. Support Care Cancer 2018; 26: 2707–2715. 29478188 10.1007/s00520-018-4100-x

[160] Rost M De Clercq E Rakic M , et al. Barriers to palliative care in pediatric oncology in Switzerland: a focus group study. J Pediatr Oncol Nurs 2020; 37: 35–45. 31478463 10.1177/1043454219871082

[161] Spencer L Battye L. Palliative care in the community for children with cancer in South East England. Eur J Oncol Nurs 2001; 5: 190–197. 12849032 10.1054/ejon.2001.0147

[162] Tosello B Dany L Bétrémieux P , et al. Barriers in referring neonatal patients to perinatal palliative care: a French multicenter survey. PLoS One 2015; 10: e0126861. 10.1371/journal.pone.0126861 PMC4433103 25978417

[163] Twamley K Craig F Kelly P , et al. Underlying barriers to referral to paediatric palliative care services: knowledge and attitudes of health care professionals in a paediatric tertiary care centre in the United Kingdom. J Child Health Care 2014; 18: 19–30. 23610238 10.1177/1367493512468363

[164] Twamley K Kelly P Moss R , et al. Palliative care education in neonatal units: impact on knowledge and attitudes. BMJ Support Palliat Care 2013; 3: 213–220. 10.1136/bmjspcare-2012-000336 24644571

[165] Vemuri S Butler AE Brown K , et al. Palliative care for children with complex cardiac conditions: survey results. Arch Dis Child 2022; 107: 282–287. 34312164 10.1136/archdischild-2020-320866 PMC8862095

[166] Verberne LM Kars MC Schepers SA , et al. Barriers and facilitators to the implementation of a paediatric palliative care team. BMC Palliat Care 2018; 17: 23. 29433576 10.1186/s12904-018-0274-8 PMC5810030

[167] Azzizadeh Forouzi M Banazadeh M Ahmadi JS , et al. Barriers of palliative care in neonatal intensive care units: attitude of neonatal nurses in southeast Iran. Am J Hosp Palliat Med 2017; 34: 205–211. 10.1177/1049909115616597 26602317

[168] Bhat K V Rao KS Vijayasekharan K , et al. Evaluating the need for integrated pediatric palliative care services in a pediatric oncology setting: a retrospective audit. Indian J Palliat Care 2021; 27: 286–290. 34511798 10.25259/IJPC_460_20 PMC8428874

[169] Chan WCH Wong KLY Leung MMM , et al. Perceived challenges in pediatric palliative care among doctors and nurses in Hong Kong. Death Stud 2019; 43: 372–380. 30015574 10.1080/07481187.2018.1478912

[170] Chong PH Soo J Yeo ZZ , et al. Who needs and continues to need paediatric palliative care? An evaluation of utility and feasibility of the paediatric palliative screening scale (PaPaS). BMC Palliat Care 2020; 19: 18. 32041616 10.1186/s12904-020-0524-4 PMC7011544

[171] Dighe M Jadhav S Muckaden M , et al. Parental concerns in children requiring palliative care. Indian J Palliat Care 2008; 14: 16–22.

[172] Doherty M Rayala S Evans E , et al. Using virtual learning to build pediatric palliative care capacity in South Asia: experiences of implementing a teleteaching and mentorship program (Project ECHO). J Glob Oncol 2021; 7: 210–222. 10.1200/GO.20.00481 PMC8081544 33555911

[173] Gu L Li ZZ Peng N-H , et al. Barriers to and facilitators of neonatal palliative care among neonatal professionals in China. Am J Hosp Palliat Med 2022; 39: 695–700. 10.1177/10499091211046236 34538119

[174] Jung HN Ju HO. Comparison of the attitudes of nurses and physicians toward palliative care in neonatal intensive care units. Korean J Hosp Palliat Care 2021; 24: 165–173. 10.14475/jhpc.2021.24.3.165 PMC10180060 37674558

[175] Jacob J Matharu JK Palat G , et al. End-of-Life treatments in pediatric patients at a government tertiary cancer center in India. J Palliat Med 2018; 21: 907–912. 29649402 10.1089/jpm.2017.0632

[176] Khanali Mojen L Rassouli M Eshghi P , et al. Pediatric palliative care in Iran: applying regionalization of health care systems. Asian Pac J Cancer Prev 2018; 19: 1303–1311. 29802691 10.22034/APJCP.2018.19.5.1303 PMC6031829

[177] Khraisat OM Alakour NA O'Neill TM . Pediatric end-of-life care barriers and facilitators: perception of nursing professionals in Jordan. Indian J Palliat Care 2017; 23: 199–206. 28503041 10.4103/0973-1075.204232 PMC5412129

[178] Laronne A Granek L Wiener L , et al. Organizational and individual barriers and facilitators to the integration of pediatric palliative care for children: a grounded theory study. Palliat Med 2021; 35: 1612–1624. 34219546 10.1177/02692163211026171 PMC10087284

[179] Laronne A Granek L Wiener L , et al. Oncologist conceptualizations of pediatric palliative care: challenges and definitions. Support Care Cancer 2021; 29: 3981–3989. 33392772 10.1007/s00520-020-05959-z PMC8164966

[180] Lin S-C Chang K-L Huang M-C. When and how do healthcare professionals introduce specialist palliative care to the families of children with life-threatening conditions in Taiwan? A qualitative study. J Pediatr Nurs 2022; 64: e136–e144. 10.1016/j.pedn.2021.12.004 34980527

[181] Endah Purnamaningsih Maria Margaretha S Mulatsih S Effendy C , et al. Qualitative analysis of family-centered care for children with cancer in palliative wards: an evaluation of needs and barriers in resource-limited settings. Open Access Maced J Med Sci 2021; 9: 1–7.

[182] Thomas P Sadasivan A Warrier M , et al. Palliative care in Duchenne muscular dystrophy: a study on parents’ understanding. Indian J Palliat Care 2021; 27: 146–151. 34035633 10.4103/IJPC.IJPC_259_20 PMC8121239

[183] Sadeghi N Hesami SA Sadeghi S , et al. Barriers to palliative care in the neonatal intensive care unit from nurses’ perspective: a qualitative study. Med Nurs J 2021; 10: 1–8.

[184] Salins N Hughes S Preston N. Presuppositions, cost-benefit, collaboration, and competency impacts palliative care referral in paediatric oncology: a qualitative study. BMC Palliat Care 2022; 21: 215. 36456939 10.1186/s12904-022-01105-0 PMC9717409

[185] Seino Y Kurosawa H Shiima Y , et al. End-of-life care in the pediatric intensive care unit: survey in Japan. Pediatr Int 2019; 61: 859–864. 31247125 10.1111/ped.13924

[186] Tapawan SJC Wang FS Lee MW , et al. Perspectives on palliative care among Duchenne muscular dystrophy patients and their families in Singapore. Ann Acad Med Singapore 2020; 49: 72–77. 32246708

[187] Yotani N Kizawa Y. Specialist palliative care service for children with life-threatening conditions: a nationwide survey of availability and utilization. J Pain Symptom Manag 2018; 56: 582–587. 10.1016/j.jpainsymman.2018.06.005 29909002

[188] Yu J Song IG Kim CH , et al. Perceptions of pediatric palliative care among physicians who care for pediatric patients in South Korea. J Palliat Med 2020; 23: 346–352. 31580756 10.1089/jpm.2018.0584

[189] Zhong Y Black BP Kain VJ , et al. Facilitators and barriers affecting implementation of neonatal palliative care by nurses in Mainland China. Front Pediatr 2022; 10: e887711. 10.3389/fped.2022.887711 PMC9263274 35813382

[190] Bradford N Irving H Murray J , et al. Paediatric palliative care services in Queensland: an exploration of the barriers, gaps and plans for service development. Neonatal Paediatr Child Heal Nurs 2012; 15: 2–7.

[191] Chang E MacLeod R Drake R. Characteristics influencing location of death for children with life-limiting illness. Arch Dis Child 2013; 98: 419–424. 23599439 10.1136/archdischild-2012-301893

[192] Evans AM Thabrew H Arroll B , et al. Audit of psychosocial and palliative care support for children having allogeneic stem cell transplants at the New Zealand national allogeneic transplant centre. Children 2021; 8: 356. 33946879 10.3390/children8050356 PMC8146388

[193] Jessop S Phelan C. Health-care workers' understanding of and barriers to palliative care services to aboriginal children with cancer. J Paediatr Child Health 2022; 58: 1390–1395. 35488850 10.1111/jpc.16000

[194] Kain V. Exploring the barriers to palliative care practice in neonatal nursing: a focus group study. Neonatal Paediatr Child Heal Nurs 2011; 14: 9–14.

[195] Kain VJ. The Praecox program: pilot testing of an online educational program to improve neonatal palliative care practice. J Neonatal Nurs 2017; 23: 188–192.

[196] Kilcullen M Ireland S. Palliative care in the neonatal unit: neonatal nursing staff perceptions of facilitators and barriers in a regional tertiary nursery. BMC Palliat Care 2017; 16: 32. 28490381 10.1186/s12904-017-0202-3 PMC5425982

[197] Monterosso L Kristjanson LJ Aoun S , et al. Supportive and palliative care needs of families of children with life-threatening illnesses in Western Australia: evidence to guide the development of a palliative care service. Palliat Med 2007; 21: 689–696. 18073255 10.1177/0269216307083032

[198] Vemuri S Baker L Williams K , et al. The last 2 years of life for children with severe physical disability: Observations from a tertiary paediatric centre. J Paediatr Child Health 2018; 54: 1357–1361. 29943874 10.1111/jpc.14092

[199] Connor S Sisimayi C Downing J , et al. Assessment of the need for palliative care for children in South Africa. Int J Palliat Nurs 2014; 20: 130–134. 24675539 10.12968/ijpn.2014.20.3.130

[200] Downing JD Marston J Selwyn C , et al. Developing children’s palliative care in Africa through beacon centres: lessons learnt. BMC Palliat Care 2013; 12: 8. 23419095 10.1186/1472-684X-12-8 PMC3584905

[201] Cuervo-Suarez MI Claros-Hulbert A Manzano-Nunez R , et al. Pediatric palliative care during end of life: a privilege of a few in a tertiary referral hospital from Colombia. Am J Hosp Palliat Care 2020; 37: 636–640. 32323561 10.1177/1049909120920542

[202] García-Quintero X Claros-Hulbert A Tello-Cajiao ME , et al. Using EmPalPed—An educational toolkit on essential messages in palliative care and pain management in children—as a strategy to promote pediatric palliative care. Children 2022; 9: 838. 35740775 10.3390/children9060838 PMC9221893

[203] Balkin EM Thompson D Colson KE , et al. Physician perspectives on palliative care for children with neuroblastoma: an international context. Pediatr Blood Cancer 2016; 63: 872–879. 26784890 10.1002/pbc.25900

[204] Bergstraesser E Hain RD Pereira JL. The development of an instrument that can identify children with palliative care needs: the Paediatric Palliative Screening Scale (PaPaS Scale): a qualitative study approach. BMC Palliat Care 2013; 12: 20. 23657092 10.1186/1472-684X-12-20 PMC3663726

[205] De Clercq E Rost M Pacurari N , et al. Aligning guidelines and medical practice: literature review on pediatric palliative care guidelines. Palliat Support Care 2017; 15: 474–489. 28065197 10.1017/S1478951516000882

[206] Delgado E Barfield RC Baker JN , et al. Availability of palliative care services for children with cancer in economically diverse regions of the world. Eur J Cancer 2010; 46: 2260–2266. 20541395 10.1016/j.ejca.2010.05.006 PMC2916078

[207] Ehrlich BS Movsisyan N Batmunkh T , et al. Barriers to the early integration of palliative care in pediatric oncology in 11 Eurasian countries. Cancer 2020; 126: 4984–4993. 32813913 10.1002/cncr.33151 PMC7981844

[208] Ehrlich BS Yakimkova T Batmunkh T , et al. Translating research to action: the development of a pediatric palliative cancer care advocacy tool in Eurasia. J Glob Oncol 2022; 8: e2100270. 10.1200/GO.21.00270 PMC8806380 35084997

[209] Friedrichsdorf SJ Remke S Hauser J , et al. Development of a pediatric palliative care curriculum and dissemination model: education in palliative and end-of-life care (EPEC) pediatrics. J Pain Symptom Manag 2019; 58: 707–720.e3. 10.1016/j.jpainsymman.2019.06.008 PMC6754756 31220594

[210] Hammond J Wool C Parravicini E. Assessment of healthcare professionals’ self-perceived competence in perinatal/neonatal palliative care after a 3-day training course. Front Pediatr 2020; 8: e571335. 10.3389/fped.2020.571335 PMC7536297 33072677

[211] Hilden JM Emanuel EJ Fairclough DL , et al. Attitudes and practices among pediatric oncologists regarding end-of-life care: results of the 1998 American society of clinical oncology survey. J Clin Oncol 2001; 19: 205–212. 11134214 10.1200/JCO.2001.19.1.205

[212] Knapp C Woodworth L Wright M , et al. Pediatric palliative care provision around the world: a systematic review. Pediatr Blood Cancer 2011; 57: 361–368. 21416582 10.1002/pbc.23100

[213] Krakauer E Al-Shammary SA Duraisamy B , et al. Palliative care models and innovations in 4 Eastern Mediterranean Region countries: a case-based study. East Mediterr Health J 2022; 27: 622–628. 36134495 10.26719/emhj.22.038

[214] McNeil MJ Ehrlich B Wang H , et al. Ideal vs actual timing of palliative care integration for children with cancer in Latin America. JAMA Netw Open 2023; 6: e2251496. 10.1001/jamanetworkopen.2022.51496 PMC9857245 36656580

[215] McNeil MJ Ehrlich BS Wang H , et al. Physician perceptions of palliative care for children with cancer in Latin America. JAMA Netw Open 2022; 5: e221245. 10.1001/jamanetworkopen.2022.1245 PMC8905380 35258577

[216] Mekelenkamp H Schröder T Trigoso E , et al. Specialized pediatric palliative care services in pediatric hematopoietic stem cell transplant centers. Children 2021; 8: 615. 34438506 10.3390/children8080615 PMC8393700

[217] Mojen LK Rassouli M Eshghi P , et al. Palliative care for children with cancer in the Middle East: a comparative study. Indian J Palliat Care 2017; 23: 379–386. 29123342 10.4103/IJPC.IJPC_69_17 PMC5661338

[218] Taylor J Booth A Beresford B , et al. Specialist paediatric palliative care for children and young people with cancer: a mixed-methods systematic review. Palliat Med 2020; 34: 731–775. 32362212 10.1177/0269216320908490 PMC7243084

[219] Weaver MS Rosenberg AR Tager J , et al. A summary of pediatric palliative care team structure and services as reported by centers caring for children with cancer. J Palliat Med 2018; 21: 452–462. 29173030 10.1089/jpm.2017.0405 PMC5915222

[220] Bluebond-Langner M Belasco JB Goldman A , et al. Understanding parents’ approaches to care and treatment of children with cancer when standard therapy has failed. J Clin Oncol 2007; 25: 2414–2419. 17557955 10.1200/JCO.2006.08.7759

[221] Together for Short Lives. A guide to children’s palliative care. Bristol: Together for Short Lives, 2018.

[222] De Clercq E Rost M Rakic M , et al. The conceptual understanding of pediatric palliative care: a Swiss healthcare perspective. BMC Palliat Care 2019; 18: 55. 31296209 10.1186/s12904-019-0438-1 PMC6625075

[223] World Health Organisation. WHO definition of palliative care, https://www.who.int/health-topics/palliative-care (2013, accessed 04 January 2023).

[224] Skivington K Matthews L Simpson SA , et al. A new framework for developing and evaluating complex interventions: update of medical research council guidance. BMJ 2021; 374: e2061. 10.1136/bmj.n2061 PMC8482308 34593508

