# Supplementary material for: Barriers and facilitators influencing referral and access to palliative care for children and young people with life-limiting and life-threatening conditions: a scoping review of the evidence
Source: Palliat Med. 2024 Sep 9;38(9):981–99. doi: 10.1177/02692163241271010 (PMC11491046; doi:10.1177/02692163241271010)
Supplement: sj-docx-1-pmj-10.1177_02692163241271010 – Supplemental material for Barriers and facilitators influencing referral and access to palliative care for children and young people with life-limiting and life-threatening conditions: a scoping review of the evidence [file sj-docx-1-pmj-10.1177_02692163241271010.docx]

Supplementary file 1: Search strategies

Medline (ovid) search strategy

| **Concept** | **Search terms** |
| --- | --- |
| Children and young people | 1 P?ediatric*.tw.  2 child*.tw.  3 infant*.tw.  4 toddler*.tw.  5 adolescen*.tw.  6 teen*.tw.  7 youth*.tw.  8 juvenile*.tw.  9 neonat*.tw.  10 newborn*.tw.  11 new born*.tw.  12 baby.tw.  13 babies.tw.  14 boy*.tw.  15 girl*.tw.  16 minors.tw.  17 exp Pediatrics/  18 exp Child/  19 exp Child, Preschool/  20 exp Infant/  21 exp Infant, Newborn/  22 exp Adolescent/  23 exp Minors/ |
| Palliative care | 24 palliat*.tw.  25 terminal care.tw.  26 hospice*.tw.  27 life limit*.tw.  28 end of life care.tw.  29 support care.tw.  30 exp "Hospice and Palliative Care Nursing"/  31 exp Palliative Care/  32 exp Terminal Care/  33 exp Hospices/ |
| Referral and access | 34 access*.tw.  35 provision*.tw.  36 uptake.tw.  37 engage*.tw.  38 attend*.tw.  39 referral*.tw.  40 refer.tw.  41 referring.tw.  42 referred.tw.  43 exp "Referral and Consultation"/ |
|  | 44 or/1-23  45 or/24-33  46 or/34-43  47 44 and 45 and 46 |

PsycINFO (Ovid) search strategy

| **Concept** | **Search terms** |
| --- | --- |
| Children and young people | 1 P?ediatric*.tw.  2 child*.tw.  3 infant*.tw.  4 toddler*.tw.  5 adolescen*.tw.  6 teen*.tw.  7 youth*.tw.  8 juvenile*.tw.  9 neonat*.tw.  10 newborn*.tw.  11 new born*.tw.  12 baby.tw.  13 babies.tw.  14 boy*.tw.  15 girl*.tw.  16 minors.tw.  17 exp Pediatrics/ |
| Palliative care | 18 palliat*.tw.  19 terminal care.tw.  20 hospice*.tw.  21 life limit*.tw.  22 end of life care.tw.  23 support care.tw.  24 exp "Death and Dying"/  25 exp Palliative Care/  26 exp Terminally Ill Patients/  27 exp Hospice/ |
| Referral and access | 28 access*.tw.  29 provision*.tw.  30 uptake.tw.  31 engage*.tw.  32 attend*.tw.  33 referral*.tw.  34 refer.tw.  35 referring.tw.  36 referred.tw.  37 exp Professional Referral/  38 exp Self-Referral/  39 exp Health Care Access/ |
|  |  |
|  | 40 or/1-17  41 or/18-27  42 or/28-39  43 40 and 41 and 42 |

Embase (Ovid) search strategy

| **Concept** | **Search terms** |
| --- | --- |
| Children and young people | 1 P?ediatric*.tw.  2 child*.tw.  3 infant*.tw.  4 toddler*.tw.  5 adolescen*.tw.  6 teen*.tw.  7 youth*.tw.  8 juvenile*.tw.  9 neonat*.tw.  10 newborn*.tw.  11 new born*.tw.  12 baby.tw.  13 babies.tw.  14 boy*.tw.  15 girl*.tw.  16 minors.tw.  17 exp Pediatrics/  18 exp Child/  19 exp Child, Preschool/  20 exp Infant/  21 exp Infant, Newborn/  22 exp Adolescent/  23 exp Minors/  24 exp "minor (person)"/ |
| Palliative care | 25 palliat*.tw.  26 terminal care.tw.  27 hospice*.tw.  28 life limit*.tw.  29 end of life care.tw.  30 support care.tw.  31 exp "Hospice and Palliative Care Nursing"/  32 exp Palliative Care/  33 exp Terminal Care/  34 exp Hospices/  35 exp palliative nursing/  36 exp palliative therapy/  37 exp terminal care/  38 exp hospice care/  39 exp hospice nursing/ |
| Referral and access | 40 access*.tw.  41 provision*.tw.  42 uptake.tw.  43 engage*.tw.  44 attend*.tw.  45 referral*.tw.  46 refer.tw.  47 referring.tw.  48 referred.tw.  49 exp "Referral and Consultation"/  50 exp patient referral/  51 exp health care access/  52 exp "Healthcare Access and Quality Index"/ |
|  | 53 or/1-24  54 or/25-39  55 or/40-52  56 53 and 54 and 55 |

CINAHL (EBSCO) search strategy

| **Concept** | **Search terms** |
| --- | --- |
| Children and young people | 1 TI p#ediatric* OR AB p#ediatric*  2 TI child* OR AB child*  3 TI infant* OR AB infant*  4 TI toddler* OR AB toddler*  5 TI adolescen* OR AB adolescen*  6 TI teen* OR AB teen*  7 TI youth* OR AB youth*  8 TI juvenile* OR AB juvenile*  9 TI neonat* OR AB neonat*  10 TI newborn* OR AB newborn*  11 TI "new born*" OR AB "new born*"  12 TI baby OR AB baby  13 TI babies OR AB babies  14 TI boy* OR AB boy*  15 TI girl* OR AB girl*  16 TI minors OR AB minors  17 (MH "Adolescence+")  18 (MH "Infant, Newborn+")  19 (MH "Infant+")  20 (MH "Minors (Legal)")  21 (MH "Child+") |
| Palliative care | 22 TI palliat* OR AB palliat*  23 TI "terminal care" OR AB "terminal care"  24 TI hospice* OR AB hospice*  25 TI "life limit*" OR AB "life limit*"  26 TI "end of life care" OR AB "end of life care"  27 TI "support care" OR AB "support care"  28 (MH "Hospices")  29 (MH "Hospice Care")  30 (MH "Hospice and Palliative Nursing")  31 (MH "Palliative Care")  32 (MH "Palliative Medicine")  33 (MH "Life Support Care+")  34 (MH "Terminal Care+") |
| Referral and access | 35 TI access* OR AB access*  36 TI provision* OR AB provision*  37 TI uptake OR AB uptake  38 TI engage* OR AB engage*  39 TI attend* OR AB attend*  40 TI referral* OR AB referral*  41 TI refer OR AB refer  42 TI referring OR AB referring  43 TI referred OR AB referred  44 (MH "Referral and Consultation+")  45 (MH "Direct Access")  46 (MH "Health Services Accessibility+") |
|  | 47 or/1-21  48 or/22-34  49 or/35-46  50 47 and 48 and 49 |

Cochrane Library search strategy

| **Concept** | **Search terms** |
| --- | --- |
| Children and young people | 1 (P?ediatric*):ti OR (P?ediatric*):ab  2 (child*):ti OR (child*):ab  3 (infant*):ti OR (infant*):ab  4 (toddler*):ti OR (toddler*):ab  5 (adolescen*):ti OR (adolescen*):ab  6 (teen*):ti OR (teen*):ab  7 (youth*):ti OR (youth*):ab  8 (juvenile*):ti OR (juvenile*):ab  9 (neonat*):ti OR (neonat*):ab  10 (newborn*):ti OR (newborn*):ab  11 (new NEXT (born*)):ti OR (new NEXT (born*)):ab  12 (baby):ti OR (baby):ab  13 (babies):ti OR (babies):ab  14 (boy*):ti OR (boy*):ab  15 (girl*):ti OR (girl*):ab  16 (minors):ti OR (minors):ab  17 MeSH descriptor: [Child] explode all trees  18 MeSH descriptor: [Infant] explode all trees  19 MeSH descriptor: [Adolescent] explode all trees  20 MeSH descriptor: [Infant, Newborn] explode all trees  21 MeSH descriptor: [Minors] explode all trees |
| Palliative care | 22 (palliat*):ti OR (palliat*):ab  23 ("terminal care"):ti OR ("terminal care"):ab  24 (hospice*):ti OR (hospice*):ab  25 (life NEXT (limit*)):ti OR (life NEXT (limit*)):ab  26 ("end of life care"):ti OR ("end of life care"):ab  27 ("support care"):ti OR ("support care"):ab  28 MeSH descriptor: [Palliative Care] explode all trees  29 MeSH descriptor: [Palliative Medicine] explode all trees  30 MeSH descriptor: [Hospice and Palliative Care Nursing] explode all trees  31 MeSH descriptor: [Hospices] explode all trees  32 MeSH descriptor: [Hospice Care] explode all trees  33 MeSH descriptor: [Terminal Care] explode all trees |
| Referral and access | 34 (access*):ti OR (access*):ab  35 (provision*):ti OR (provision*):ab  36 (uptake):ti OR (uptake):ab  37 (engage*):ti OR (engage*):ab  38 (attend*):ti OR (attend*):ab  39 (referral*):ti OR (referral*):ab  40 (refer):ti OR (refer):ab  41 (referring):ti OR (referring):ab  42 (referred):ti OR (referred):ab  43 MeSH descriptor: [Health Services Accessibility] explode all trees  44 MeSH descriptor: [Referral and Consultation] explode all trees |
|  | 45 or/1-21  46 or/22-33  47 or/34-44  48 45 and 46 and 47 |
