# Supplementary material for: Barriers and facilitators influencing referral and access to palliative care for children and young people with life-limiting and life-threatening conditions: a scoping review of the evidence
Source: Palliat Med. 2024 Sep 9;38(9):981–99. doi: 10.1177/02692163241271010 (PMC11491046; doi:10.1177/02692163241271010)
Supplement: sj-docx-2-pmj-10.1177_02692163241271010 – Supplemental material for Barriers and facilitators influencing referral and access to palliative care for children and young people with life-limiting and life-threatening conditions: a scoping review of the evidence [file sj-docx-2-pmj-10.1177_02692163241271010.docx]

| **Evidence source Details and Characteristics** |
| --- |
| Citation details *(first author, year of publication, title, journal)* |
| Country of origin |
| Context |
| Study Aims |
| Study Design (Prospective, Retrospective, Longitudinal or Cohort study) |
| Research Methodology & Methods |
| Participant Details *(age and sample size)* |
| **Details/Results extracted from source of evidence (in relation to the concept of the scoping review)** |
| Type of barriers and/or facilitators affecting referral to and uptake of paediatric palliative care for children and young people and associated levels of the Socio-Ecological framework (1) Individual; 2) Interpersonal; 3) Organisational; 4) Community; 5) Society) |
| Details of interventions to enhance referral for children and young people to palliative care and associated levels of the Socio-Ecological framework (1) Individual; 2) Interpersonal; 3) Organisational; 4) Community; 5) Society) |

Supplementary File 2: Data extraction tool
